# Role of Callous and Unemotional (CU) Traits on the Development of Youth with Behavioral Disorders: A Systematic Review

**DOI:** 10.3390/ijerph18094712

**Published:** 2021-04-28

**Authors:** Myriam Squillaci, Valérie Benoit

**Affiliations:** 1 Department of Special Education, University of Fribourg, 1700 Fribourg, Switzerland; 2 Department of Special Education, University of Teacher Education of State of Vaud, 1014 Lausanne, Switzerland; valerie.benoit@hepl.ch

**Keywords:** callous-unemotional traits, childhood, functioning, review

## Abstract

Numerous studies have shown that youth with behavioral disorders (BD) present an increased risk for developing severe and persistent antisocial behaviors in adulthood. Retrospective research notes that not all children and adolescents follow a negative trajectory and explains this heterogeneity in particular by the severity of CU traits. Our study examines how these traits affect the functioning of children and adolescents with BD. Method: A systematic literature review conducted through various databases and using different keywords made it possible to analyze 52 studies published from 2015 to 2020 that measured the bidirectional effects of CU traits on the functioning of young. Results: Out of the 52 studies, 47 analyzed links between CU traits and neurobiological or mental health, 20 examined family and school contexts, eight focused on social adjustment, 10 on social interactions and 19 measured links with cognitive functioning, especially executive functions. Conclusion: Consistent with previous recommendations in the field, our findings emphasize the importance of assessing the presence of UC traits in early childhood to prevent the emergence of comorbid disorders and to target multimodal (early) interventions to influence the life trajectories of youth with high CU traits.

## 1. Introduction

Behavioral disorders (BD), including disruptive, aggressive, and/or antisocial behavior, are some of the most common disorders in children and adolescents [1]. The notion of BD used in our review is an umbrella term, including children and adolescents with a range of emotional and behavioral disorders. It includes a population with heterogeneous behavioral diagnoses such as oppositional defiant disorder (ODD), conduct disorder (CD), internalized disorders (ID), conditions often associated with aggressive and/or antisocial behavior [2]. These patterns place a very significant burden on the individual as well as on society in general. Numerous studies have shown that youth with DB present an increased risk for developing severe and persistent antisocial behaviors in adulthood, such as being involved in substance abuse, crime, unemployment, and early death [3,4]. Retrospective research in the field notes heterogeneous developmental trajectories that could be explained by certain risk factors, including the severity of callous-unemotional (CU) traits, a constellation of emotional and personality traits in children considered as a precursor to adult psychopathy [5]. Children with CU traits show a behavioral pattern characterized by a lack of empathy and guilt, insensitivity to others’ feelings, shallow and deficient affect (e.g., lack of emotion recognition, of perspective-taking), unconcern for performance, and the callous use of others for one’s own personal interest [1,6,7,8,9].

Despite Frick’s pioneering work [10,11,12], the issue of CU traits in children and adolescents emerged officially only with the fifth revision of the Diagnostic and Statistical Manual of Mental Disorders (DSM-5) of the American Psychiatric Association [1]. Previously limited to the conceptualization of psychopathy in adulthood, these traits now designate a subtype of youth with (a) a severe and recalcitrant form of antisocial behavior and (b) distinct neurological, cognitive, emotional, and social characteristics [6] (p. 710). CU traits are constitutive of the “Limited Prosocial Emotions” (LPE) specifier of the conduct disorder [1]. This designation has major implications in terms of etiology, development, and intervention and should therefore be considered in the assessment of behavioral disorders, in particular conduct disorder.

The following sections summarize CU traits assessment and these three domains, mostly based on a review of literature reviews on CU traits in children and adolescents. Indeed, research on CU traits in children and adolescents has been particularly flourishing this past decade.

### 1.1. CU Traits Assessment

Several instruments exist to measure CU traits in youth (e.g., Youth Psychopathy Traits Inventory; Psychopathy Checklist: Youth Version; Child Problematic Traits Inventory [13]). However, a commonly used instrument for assessing CU traits in children and adolescents is the Inventory of Callous-Unemotional Traits (ICU) [14]. This scale, which has contributed significantly to the development of the LPE specifier introduced in the DSM-5 [15], consists of 24 items divided into three dimensions (callous, uncaring, and unemotional). Responses are scored on a 4-point Likert scale, ranging from 0 (not at all) to 3 (definitely true) [15]. As CU traits assessment requires multiple informants (the child himself, his caregivers, and/or teachers [1]), self-report and other reports of the ICU exist. Psychometric properties of the ICU such as factorial structure and internal consistency have been explored by, among others, Kimonis et al. [16] in the USA, Essau et al. [17] in Germany, Ciucci et al. [18] in Italy, Roose et al. [19] in the Netherlands, Pechorro et al. [20] in Portugal [20] and Ezpeleta et al. [21] in Spain. To our knowledge, to date no version of the ICU scale has been translated and validated in French-speaking language, despite Garcia et al. [13] announce of actual work on French validation of CU traits’ scales. Several validation studies have shown certain robustness of its three-dimensional structure regardless of language, age, sex, and evaluators, even if fit indices seem to be modest and reach acceptable fit with post hoc modification to the model [6] (p. 714), which is known to be questionable [22]. The recent meta-analysis by Cardinale and Marsh [15] reports good internal consistency and strong convergent and external validity for all scores on the ICU scale and the callous and uncaring subscales, but not for the unemotional scores [15], as previously noted by Kimonis et al. [16]. Findings available to date support the usefulness of this scale, mainly at the total score level [23]. Strong relations with variables linked to antisocial behavior have been established by several studies, e.g., [6,24].

### 1.2. Etiology

Recent research shows that children and adolescents with CU traits present specific genetic, cognitive, emotional, biological, and environmental characteristics, some of which being similar to those of adults with psychopathy [25,26]. This implies that the etiological factors underlying their behavioral problems are different from other young people with severe BD [24]. Both individual (e.g., genetic, neurobiological) and environmental risk factors contribute to the risk of developing psychopathic traits [26,27,28,29]. In this respect, the difficulty of isolating purely endogenous risk factors can be noted since they are in constant interaction with environmental factors (i.e., passive/evocative gene-environment correlation phenomenon (rGE), e.g., [26]). Nevertheless, the following is an attempt at a taxonomic presentation intended to provide a better understanding of the origin and effects of CU traits on youth functioning.

#### 1.2.1. Genetic Risk Factors

Despite possible neurocognitive vulnerabilities that enhance susceptibility to develop psychopathic features, Viding and McCrory [26] have clearly stated that to date, there are no genes identified as being linked to the development of psychopathy. Thus, although an individual’s genome probably limits a range for phenotypic expression, it does not specify in advance how an individual will evolve, as development is strongly influenced by environmental factors [26,28] (p. 569). If there is currently insufficient evidence to question specific genetic mechanisms, the review conducted by Moore et al. [30] indicates an estimated 36% to 67% heredity of CU traits. To date, heredity is thought to influence primarily disruptive behaviors with high levels of CU traits, whereas environmental factors are thought to have a greater impact on disruptive behaviors with low levels of CU traits [26]. In this regard, research results, including twin samples, indicate that the stability of CU/psychopathy traits is largely determined by genetic influences [26].

#### 1.2.2. Emotional and Cognitive Risk Factors

The “Limited Prosocial Emotions” specifier states that children and adolescents with CU traits have an emotional deficit without indicating which particular deficit is involved [31]. Several authors agree that low interpersonal emotional sensitivity and fearlessness play a major role in CU traits, notably their interaction, e.g., [6,32]. These characteristics are further exacerbated by environmental risk factors as harsh parenting practices and lack of parental warmth [32]. Low interpersonal emotional sensitivity would affect the development of emotional empathy from the age of 2 (i.e., diminished facial or verbal expressions of concern for the distress of others, impaired eye contact), e.g., [6,32]. As for fearlessness, it could have an impact on the development of behavioral inhibition in response to both social (e.g., punishment) and non-social (e.g., mentally appropriate frightening stimuli) threats. As a result, children would be less likely to learn to relate fear or other aversive emotions with actions that involve risk, harm, or punishment [32] (p. 14). Thus, these youths would also take greater risks compared to children with behavioral problems without CU traits [31]. In terms of cognitive risk factors, some reviews indicate a negative correlation of intellectual quotient (IQ) and CU traits [31], as well as impaired decision-making and impaired perception of the reward processing [31,33]. These emotional and cognitive characteristics can be explained in part by neurobiological factors.

#### 1.2.3. Neurobiological Factors

Research using functional (fMRI) and/or structural (sMRI) brain imaging provides a better understanding of the neural mechanisms involved in the etiology of CU traits, although results obtained in children and adolescents are less consistent than in adults [25,34]. Abnormalities in different parts of the brain seemed involved [28], particularly the prefrontal and limbic structures, including the amygdala and striatum [25,34,35]. Those areas are involved in the production of prosocial behavior and in learning the relationship between a behavior and its consequences. Thus, the amygdala plays fundamental roles in recognition of (mostly negative) emotions and in reactions, especially to fear stimuli [25]. For example, findings indicate that compared to typically developing (TD) youth or youth with CD without CU traits, young with CD and high CU traits would have hypo-reactivity of the amygdala to fear stimuli, partly explaining their proactive, aggressive behaviors. On the contrary, young with CD and low CU traits would show hyper-reactivity of the amygdala to fear stimuli, partly explaining their difficulty in regulating their emotions and their reactive aggressive behaviors when they feel threatened [33]. With respect to the prefrontal cortex, it is involved in many functions such as executive functions (EF), impulsivity, inhibition, sensitivity to punishment and reward, risk-taking, and decision-making (op. cit.). Failing executive functions or disinhibition/impulsivity may also explain how high CU traits lead to more severe forms of antisocial behavior over the life course [36] (p. 14). Research in the field also points out that youth with BD would present different brain functioning according to their level of CU traits and would therefore have distinct neurocognitive vulnerabilities [33].

Functional (and possibly structural) impairments at these levels could be associated with antisocial, callous and unemotional behavior, and adult psychopathic traits could have juvenile origins (i.e., similar deficits are observed in adult neuroimaging research [25]). However, a causal model cannot be deduced from this without risking stigmatizing children and adolescents who are still developing [25]. Thus, while functional brain deficits may contribute to a better understanding of the etiology of antisocial behavior and CU traits in children and adolescents, they should be considered as part of a constellation of both endogenous and exogenous risk factors and from a biopsychosocial perspective (person-by-context interactions).

#### 1.2.4. Environmental Risk Factors

Numerous studies examined the influence of the context in the development of young with CU traits, including parental attachment quality, parental practices and interactions, parental socioeconomic status, or other life experiences such as teachers or peer relationships, e.g., [15,32,37]. For example, it has been shown how certain parenting practices, such as high levels of positive reinforcement from adoptive mothers, buffer the effects of hereditary risk for CU behaviors [38,39]. Although parenting is thus likely to be a direct, non-hereditary influence on the development of CU behavior, it interacts with aspects of children’s temperament to exacerbate or buffer the risk of CU behavior [29] (p. 123). A better understanding of the causal patterns of these children and adolescents allows for more targeted prevention [28] in order to develop certain social skills, such as empathy or emotional regulation [40], if possible, before serious disruptive behavioral problems emerge [24,41].

### 1.3. Development

Several studies have examined the influence of CU traits on one or another aspect of development, particularly on social functioning. Results have shown that, in this highly antisocial context of the sample, high levels of CU traits are significantly related to minimizing the consequences of aggression and empathy toward others. For example, it was found that young people who use forms of emotional or physical violence against a parent (child-to-parent aggression) at an early age (10 and 11 years) are more likely to have CU traits [42]. Again, some authors have found differences according to the level of CU traits. For instance, according to the model of Kuay et al. [43], adolescents with low levels of CU traits would perpetrate aggression toward their parents primarily in response to harsh parenting practices (revenge goal), while youth with high levels of CU traits would commit these acts more broadly, both toward their parents and extra-family members (i.e., peers, siblings) and rather for personal gain and dominance. CU traits are also reported to be significantly and positively associated with the perpetration of bullying at school [44,45]. Similarly, while young offenders with CU traits do not appear to be influenced by the delinquent behavior of their peers, their delinquent behavior tends to strongly influence the delinquent behavior of their friends [46]. In addition, high levels of psychopathic traits, low levels of empathy, and emotional intelligence are potential risk factors for gang membership. However, contradictory research findings prevent conclusions from being drawn regarding the influence of psychopathy and CU traits on gang membership [47].

At first glance, one might think that CU traits only influence people’s social functioning (e.g., social relationships and emotional regulation). Meanwhile, research findings in the field show the influence of these traits on other developmental domains, such as health, cognition, and context. With regard to health, it was broadly noted that CU traits are associated with lower levels of neuroticism (e.g., anxiety, fear, guilt, depression) [24]. To obtain a better understanding of the heterogeneity of conduct disorders (CD), Fanti [48] focused on the role of different physiological systems. In particular, this review showed that the dysfunctional activity of facial electromyography, which is related to reduced empathy, was more evident in the group of young people with conduct disorder and CU traits than in other subgroups (e.g., with internalized disorders). In addition, the serotonin and oxytocin systems may also play a role in the characteristics of CU traits [30]. Young with CU traits are also reported to have low levels of cortisol [35]. Low cortisol levels are generally associated with aggressive behaviors, particularly when these are early or proactive. In contrast to adults with psychopathy, high testosterone levels have so far not been demonstrated in young people. However, higher levels of dehydroepiandrosterone, a testosterone precursor, have been found in young people with antisocial behavior [35].

Regarding cognition, studies have looked at the link between reactive aggression, typical of youth with low levels of CU traits, and deficits in social information processing, impacting decision-making. Young people with such deficits in social information processing would be unable to provide appropriate responses in social situations, leading them to respond with aggressive behavior. These children would therefore treat social information differently from their non-aggressive peers [43]. Young people with high levels of CU traits would also show deficits in social information processing (SIP), related to a type of proactive aggression. In this case, the use of aggression would be more a reasoned choice than the result of a lack of anger control, particularly with the aim of dominating others [43]. Dysfunctions in terms of moral judgments (perspective-taking, SIP) and emotional reactivity thereby confer a risk of aggression throughout adolescence and into adulthood [49].

As explained in the etiology section, person-by-context interactions play a role in the development of CU behaviors, including interactions between child temperament and parenting practices [36]. For example, inconsistent, cold, and harsh parenting practices have been shown to be involved in the development and even the maintaining of CU traits [31]. However, a child with CU traits is also more likely to elicit negative parental responses and to provide parents with fewer opportunities for compliments or rewards, increasing the likelihood that the pattern of parent-child interaction becomes largely negative [26]. However, studies have shown that parenting behavior may mitigate or exacerbate certain child traits such as fearlessness or even certain genetic characteristics, which themselves are associated with the risk of developing CU behaviors [36,39].

In light of the above, it is not surprising that some authors such as Waschbusch et al. [5,50] or Frick and Ray [6] argue that CD children with high levels of CU traits have a different developmental pathway than CD children without these traits. Characteristics associated with CU traits, different from TD children or children with CD without CU traits, are thought to appear early in development [6]. Early childhood studies report that CU behaviors observed at the age of three are correlated with deficits in empathy, guilt, and awareness [51]. They predict later aggression, concurrent and future behavioral problems, and lower social acceptance by peers [29,52]. At risk for chronic BD during childhood [36], children and adolescents with CU traits appear to be at risk for more severe and persistent antisocial consequences [13,24], even taking into account the severity of their conduct problems, the age of onset of these problems and common comorbidity problems (e.g., attention deficit disorder with or without hyperactivity (ADHD), anxiety disorders) [53]. CU traits represent a significant risk factor for entering future trajectories of antisocial and aggressive behavior [54] or even developing an antisocial personality disorder or delinquent or even criminal behavior in adulthood [13,55].

In addition, CU traits are reported to be stable over time [51], both from early childhood to late childhood [54] and from childhood to adolescence [24]. However, half of the studies reviewed by Wilkinson et al. [56] show that young people with CU traits may respond positively to some interventions. This means that CU traits, such as any personality trait or other psychopathology, are not fixed: they may decrease following certain interventions (see also [28,37]). Thus, although the developmental trajectory of these children and adolescents differs from that of young people without CU traits, it is no less heterogeneous (principle of equifinality, e.g., [24,28]).

### 1.4. Intervention

Research has shown that children and adolescents with both severe behavior problems and high CU traits tend to respond less positively to standard interventions [57]. In particular, they are reported to be insensitive to punishment [6]. Nevertheless, they are reported to show positive responses to some intensive interventions tailored to their unique emotional and cognitive characteristics [53]. The review by Lui et al. [9] has indeed highlighted the interest of interventions in emotion recognition and perspective-taking for adolescents with BD, although young people with antisocial behaviors were poorly represented in the studies reviewed. Most programs targeting the development of perspective skills show positive outcomes, which is also promising for young people with CU traits to work on their emotional skills [9]. These results are thus in line with the recommendations of Baker et al. [33] to intervene intensively in developing sensitivity to other people’s distress cues, improving prediction errors, and identifying expected value signals when making decisions.

Still, according to Baker et al. [33], youth with low levels of CU traits are likely to respond to interventions that focus on improving the management of emotions, particularly anger. Thus, computer-based emotion recognition training programs [58] or programs that address deficits in reciprocal eye contact that can modify emotional engagement and, therefore, the quality of relationships between children and their parents seem promising, although their effectiveness remains to be demonstrated [57]. A growing number of studies report the effectiveness of certain intervention programs, including multimodal and comprehensive approaches that combine CBT, medication, family therapy, parenting interventions, and the development of socio-emotional skills, e.g., [34,51]. In relation to pharmacological intervention, Pisano et al. [51] point out that no specific treatment exists for CU traits and that while pharmacotherapy can be an interesting added value to psychosocial intervention in disruptive behavior disorders, it would improve aggression and emotional dysregulation, but not CU traits (see also Masi et al., [59]).

In this regard, the value of early intervention in preventing the development of persistent antisocial behavior has been widely noted, e.g., [29,57] particularly because early childhood, such as the prenatal stage, is a developmental period that is particularly sensitive to environmental influences [24,53,60]. Indeed, during early childhood, experience-dependent neuroplasticity is at its peak [61]. Early intervention can influence both neurodevelopment (e.g., with nutrition, including omega-3 supplementation, e.g., [25,62], or zinc [63]) and biology through psychological processes (e.g., hormone levels are influenced by interactions between parent and children [34,64] (p. 303)). Along the same lines, some research highlight the role of oxytocin, which is recognized as the main moderator of complex social behaviors (e.g., attachment, social recognition, aggression) [35]. Like social support, oxytocin (and particularly their combination) is thought to have a positive effect on stress responsiveness, thereby lowering cortisol levels. As the amygdala appears to be the preferred target of oxytocin, the effects of its administration as a treatment are promising but have still to be investigated [35]. In addition, some youth with high CU traits would also be particularly sensitive to warm parenting practices and would respond positively to parenting interventions [37,57] or positive reinforcement strategies [28,65]. Primary prevention strategies (e.g., positive reinforcement of prosocial behaviors) in preschool also are of particular interest, especially if it is implemented universally so that all children can benefit [28].

These findings demonstrate that CU traits are evolving and remind us of the importance of considering them from both a dynamic developmental perspective and a biopsychosocial approach: “If a child’s general antisocial behavior improves following intervention, changes in parental response may follow, which, in turn, could also facilitate reductions in CU traits over time” [56] (p. 560).

### 1.5. Research Aims and Questions

Since the introduction of CU traits as a conduct disorder specifier by the APA in 2013, more than 50 literature reviews about CU traits in children and adolescents have been published (e.g., Web of Science, 2013–2020). Although most of the reviews cover several aspects, they mainly study and discuss (a) diagnostic legitimacy, e.g., [6,24,66]; (b) assessment, including the validity of measurement instruments, e.g., [15,23]; (c) etiology, e.g., [33,35]; (d) developmental trajectories and outcomes, e.g., [28,51] and (e) intervention, e.g., [28,67]. Even flourishing literature review on CU traits (see above), to date and as far as we know, no review deals about links between CU traits and each dimension of human functioning. Thus, our study examines the links between CU traits and developmental aspects of young people with severe BD using the AAIDD model of human functioning [68] to highlight implications for intervention. Indeed, the AAIDD model identifies five components (Health, Context, Adjustment, Interactions, Cognition) that influence human functioning through the support provided to individuals. Defined as the set of resources and strategies that promote a person’s development, education, interests, and well-being and that improve human functioning [68], this model acts as a mediator between the components and human functioning and may concern both persons with and without intellectual disabilities. Therefore, by analyzing the effects of CU traits on each of these components, this systematic literature review aims to (1) identify how CU traits affect the functioning of children and adolescents and (2) better identify the supports to be offered to these youths and their environment.

## 2. Materials and Methods

Literature searches of English-language, peer-reviewed studies published from 2015 to 2020 were conducted in Web of Science, OvidSP, and EBSCOhost. Key terms used in searches included callous-unemotional OR callous-unemotional traits OR CU traits AND conduct disorder OR disruptive disorder OR oppositional disorder OR ODD OR CD OR behavior disorder AND social participation OR interactions OR communication OR language OR intellectual OR cognitive OR IQ OR executive function OR making decision OR theory of mind OR comorbidity OR mental health OR emotion OR regulation OR physical health OR cortisol OR frontal OR prefrontal OR orbitofrontal AND school OR family OR parents OR teachers OR trajectory OR effects. By including various clinical samples, we considered the heterogeneity in youth with CU traits such as previous reviews or meta-analyses. Each study included one or more samples in which the presence of CU traits was measured during childhood and/or adolescence by a standardized tool. The literature searches returned 250 results, and 52 publications remained for examination in line with the inclusion criteria.

**Figure 1:**
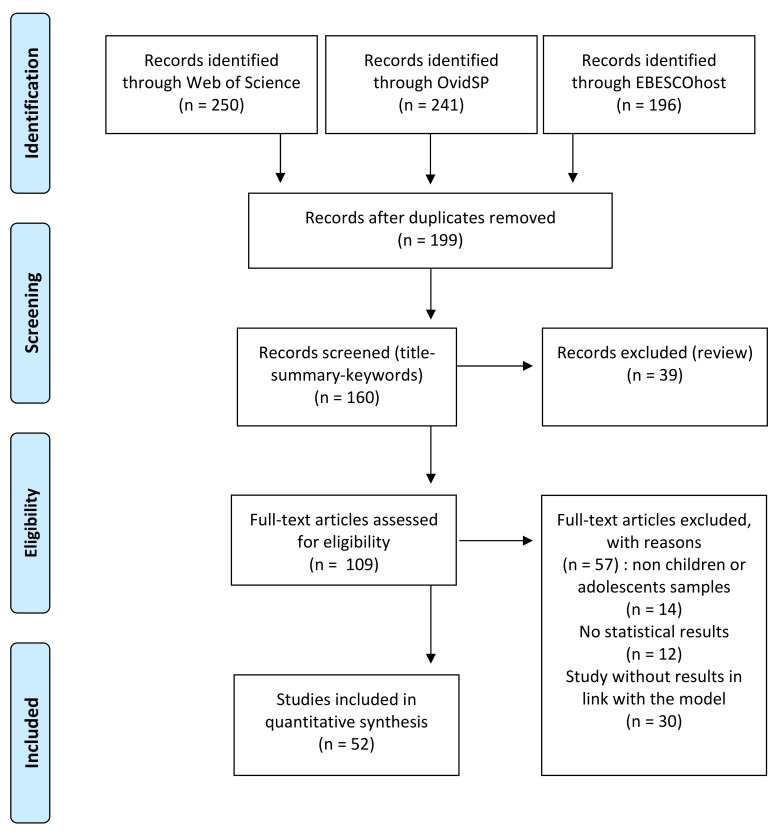
PRISMA flow diagram.

Figure 1 illustrates the article selection process and shows the total number of records from our information search. In a first step, duplicates and articles written in a language other than English, French, or Italian were removed, resulting in 199 studies. We then reviewed the titles and abstracts of all these records according to our inclusion criteria. Some full-text articles were then excluded (n = 57) because a more detailed assessment revealed, for example, that the sample was not relevant (n = 14) or that no primary data were reported (n = 13) or that no result in line with at least one dimension of the model (n = 30). The full study selection procedure resulted in 52 studies that met all inclusion criteria.

Data were coded by the two authors (and a master-level student) to ensure the reliability of the reported data and to identify discrepancies. Identified errors (12%) were then re-coded by the master-level student and one of the authors to achieve 100% agreement. To be analyzed in the review, articles were selected on the eight following inclusion criteria (partially based on the Standard Quality Assessment Criteria for Evaluating Primary Research Paper): (1) research question sufficiently described; (2) study design evident and appropriate; (3) inclusion of a sample of children or adolescents sufficiently described; (4) have measured CU traits using a validated scale; (5) the method clearly described; (6) Have measured the effects of CU traits in at least one of the five dimensions of the AAIDD model; (7) outcomes well described and statistical results reported; and (8) have been published from 2015 to 2020. The causes of exclusion were as follows: (1) sample over 20 years old; (2) articles published before 2015; (3) articles reporting narrative results; and (4) systematic reviews and meta-analyses. These inclusion criteria were used for two reasons. First, it allowed the review to focus on only the most current research published since the comprehensive review of Frick et al. [53]. Second, it enabled the analysis of only studies published after the advent of DSM- 5, the first diagnostic manual to incorporate CU traits as psychopathological characteristics.

Results were coded using a Microsoft Excel spreadsheet including 64 categories related to the preferred reporting items for systematic reviews and meta-analyses (PRISMA) (data available on request): title, years of publication (from 2015 to 2020), country, abstract (and keywords), introduction (aim of the study), methods, instruments to assess CU traits, CU traits scores, characteristics of the sample (group test, group control, age of the youth, mean age, sex, developmental characteristics, intervention: duration, setting, procedure), results (statistical results reported) on at least one of the five dimensions of the AAIDD model: mental health (14 categories: CU, comorbidity, externalizing behavior, internalizing behavior, empathy, emotion, anger, fear, etc.), neurophysiological health (8 categories: white matter, amygdala, oxytocin, cortisol, etc.), context (11 categories: school context, relationship with teachers, family context, relationships with caregivers, maternal sensitivity, supervision, etc.), social adjustment (2 categories: social competence, substance abuse), social interactions (3 categories: interactions with peers, trajectory, social roles), cognitive dimensions (8 categories: IQ, executive functions, language disorders, school performances, etc.) and two individual factors (sex, age), discussion (interpretation, bias). This article presents only data related to the main research objectives without including statistical results. This choice was made necessary by the very heterogeneous research designs in the studies, the lack of available statistical data in some dimensions of studies (e.g., some authors reported effects between CU traits and anxiety disorders by only reporting the p-value, while others provided sufficient details). Thus, the choice of a narrative review seemed to be the most appropriate way to report the effects of CU traits on development.

## 3. Results

Table S1 in Supplementary Materials provides an overview of the 52 studies published from 2015 to 2020, included in the review for analysis. Among the 52 studies, most authors reported results on more than one dimension of the model, e.g., [69,70], 47 reported effects on mental health, e.g., [71,72,73] or on neurobiological features, e.g., [69,74,75], 22 on social contexts, e.g., [36,76], eight on social adjustment, e.g., [77,78], 10 on social roles and social interactions, e.g., [79,80] and 19 on executive functions or cognition, e.g., [81,82]. The majority (k = 17) were carried out in the USA, e.g., [83,84], two in Canada [70,85], two in Australia, two in Israel, the one in Europe (Spain, Germany, Netherlands, etc.). Four studies were conducted in Switzerland in partnership with other countries, e.g., [82,86]. The review did not allow to identify any study, including a French-speaking sample.

### 3.1. Sample

The 52 studies included in the review identified CU traits in samples of youth with different behavioral (ODD, CD) or neurodevelopmental disorders (ADHD, ASD). Sample sizes differed significantly among studies (N = 23 in the study of Kimonis [87] to N = 8958 in the study of Takahashi [88]) depending essentially on the study design. One study examined monozygotic twin families [36]. The age of the youth is between early childhood (6 months) to late adolescence (18 years old), with a mean age of 10.63 years. Studies generally indicated that boys with CU traits are more likely to experience developmental difficulties than girls. The ratio of girls to boys (65% boys; 35% girls) should be assessed with caution, as several studies included only samples of boys in their studies [69,75]. This caution is all the more relevant since, in studies including similar numbers of girls and boys, the number of girls was proportionally higher in the control group than in the experimental one. The authors reported differentiated results according to the intensity of CU traits (high-low). Our results emphasize, in particular, the effects of high CU traits on young people’s functioning.

### 3.2. Studies Design and Key Methods

Table S2 highlights the instruments used to measure CU traits and developmental characteristics of the samples, as well as the methods and designs applied. Studies used various diagnostic tools validated on the DSM nosographic criteria and allowing to measure CU traits and behavior disorders. The Inventory of Callous-Unemotional Traits (ICU; [14,16,17]) is the most common scale used to measure CU traits. Despite the APA recommendations to assess CU traits in children and adolescents by multiple informants, most studies used solely one source: parent or caregiver using the parent-report questionnaire (n = 23), the child or adolescent itself using the ICU self-report (n = 13), the teacher report (n = 6) and one study measured CU traits with a counselor. Indeed, only nine studies used different informants to assess CU traits, mainly the self-report and the parent form (n = 6). None crossed three sources at a time. To assess psychopathic tendencies in youth, studies also used the Antisocial Process Screening Device (APSD) [89], in particular the callous-unemotional subscale, e.g., [88] and the narcissism and impulsivity subscales, e.g., [88], the Youth Psychopathic Traits Inventory (YPI; [90]), or the Childhood Psychopathy Scale (CPS, [91]). One study also used some items of the Strength and Difficulties Questionnaire (SDQ; Goodman, 1997) to assess CU traits [88], and another used the Preschool Forms of the Achenbach System of Empirically Based Assessment (ASEBA, [92]) to assess early CU behaviors. Finally, Crum et al. [70] used the CU subscale of the Nova Scotia Modified IOWA Conners (NSIC; [93]) as the ICU or the APSD were not published at the time of research [70].

Among many different tools (see note to Table S2), measures of behavioral and emotional symptomatology in children and adolescents (e.g., CD, ODD, ADHD, anxiety, depression) have been mainly assessed using the SDQ (e.g., [4,79]), the CBCL (e.g., [72,94]) the Kiddie-Schedule for Affective Disorders and Schizophrenia (K-SADS-PL; [95]), e.g., [96,97] and the Reactive-Proactive Aggression Questionnaire (RPQ; [98]), e.g., [69,99]. To measure the presence of autistic traits in children and adolescents, different scales have been used, as the Social Responsiveness Scale (SRS; [100]), e.g., [101] and the Social Communication Questionnaire (SCQ; [102]), e.g., [75]. Some studies also used clinician-administered interviews to assess or confirm the clinical diagnosis (ASD, CD, ODD), using tools such as the Diagnostic Interview Schedule for Children, Adolescents, and Parents (DISCAP; [103]), e.g., [84], or observational assessment, e.g., [104]. At last, we can still note that 17 studies-controlled IQ (>80 or >75) using an individually administered test of intelligence, mainly using Wechsler scales. IQ is usually used as a sample criterion for study inclusion or to match sample subsets.

Variable such as self-regulation, empathy, social competence, student-teacher relationship quality, or academic performance were measured with various tools, as listed in Table S2. This table also highlights the different study designs and key methods and shows that most studies are cross-sectional. Moreover, one-third of all selected studies (19/52) used a longitudinal design with two to six assessment points, ranging from six months to nine years, e.g., [4,88].

### 3.3. Mental Health

For many years, researchers have been investigating emotional features associated with CU traits in order to better understand the functioning of children with CD. Table S3 summarizes 36 studies that have investigated the effects of CU traits on children and adolescents’ mental health. Such relations have been found using different tools and methods: self-reported questionnaires, observations, interviews, standardized tools, hormonal test (salivary, hair), functional magnetic resonance imaging (fRMI), diffusion tensor imaging (DTI), etc. Results indicate four important outcomes: (1) all studies that have measured relationships between CU traits and externalized disorders found significant links, especially with ODD, CD, e.g., [105,106], and with ADHD or ASD [75]. As compared with children with only ODD or CD, children with ODD or CD and high CU traits exhibit antisocial behavior more severe, stable, and varied in nature [107]; (2) comorbidities with internalized disorders and anxiety disorders were also found in most studies, e.g., [41,108]; only two did not find a significant relationship with high CU traits [4,69]; (3) out of the 18 studies that investigated emotional features associated with high CU traits, 15 highlighted difficulties in emotional regulation, e.g., [101,109] while only three studies found no link [72,107,110]. Research points out the problems of emotional regulation experienced by children with high CU traits, particularly in terms of anger, sadness, and lack of empathy, and emotional overmodulation [111]. Current emotional models show an increase in the intensity of aversive and negative responses as a result of these biased assessments [107]. Outcomes of Erdogan [72] and Dackis [105] underlines that while the presence of CU traits without anxiety is characterized by difficulties in responding emotionally to the distress of others and a poor response to stress, the combination of high levels of CU traits and anxiety is characterized by negative emotionality, impulsivity, hyperarousal, strong arousal reactivity to emotional stimuli, and strong fear reactivity, driven by environmental adversity (violence of context, trauma experience, abuse); and (4) CU traits are not specific to children with CD but may also be observed in children with other disorders as ASDs or ADHD [104].

### 3.4. Neurobiological Markers and CU Traits (Physical Health)

For many years, researchers have been investigating neurobiological markers to explain the pathophysiology and symptomatology of CU traits in populations with BD. Table S4 summarizes 19 studies that investigated the links between CU traits and neurophysiological health. Such relations have been found using a number of different tools and methods such as salivary tests to assess levels of cortisol or oxytocin, heart rate, diffusion tensor imaging (DTI) to measure white matter (WM) alterations, magnetic resonance imaging (MRI) to measure brain connectivity and amygdala subregional network. Five studies (5/19) focused on hormone levels or on the autonomic nervous system. Results indicate two main findings: (1) three studies [75,79,94] compared levels of three key hormones (oxytocin, cortisol, and testosterone) in adolescents with BD and in typically developing youth (TDI), and results in related hormone levels to the severity and subtype of aggression and callous-unemotional (CU) traits [75,94]. Bakker-Huvernaars’ work [75] point out their roles in social adjustment as important regulators of complex social cognition and related behavioral responses. Oxytocin is associated with various social adjustment factors and appears to be involved in the emotional dysfunctions of different disorders such as ODD, CD, ADHD, ASD, and CU traits. Links between low levels of cortisol (and therefore a low tonic activity of the stress axis) induce social indifference, reduced cardiovascular reactivity to stress, and aggressive behavior [79]. This lack of inhibitory effects of cortisol reactivity was especially significant in boys; and (2) two other works focusing on autonomic nervous system activity highlighted underline low resting heart rate as one of the most robust and well-replicated biological markers of antisocial behavior [109]. Confronted to pleasant, neutral, or unpleasant images, children with CU traits tend to present a kind of emotional overmodulation confirmed by heart rate measures [105].

The other studies (14/19) of this dimension focused on brain dysfunctions or neural connections. Results indicate three main findings: (1) BD and CU traits would be associated with structural and functional brain abnormalities in processes subserving emotion processing and regulation [74]. Importantly, Aghajani et al. [69] demonstrated that juveniles with CD/CU traits present disorganized amygdala networks, which were accompanied by amygdala structural defects; Raschle et al. [74] found a significant positive correlation between CU traits and bilateral anterior insula volume in boys; (2) another imaging study conducted by White et al. [112] showed a reduced amygdala-ventromedial prefrontal cortex connectivity during high provocation; (3) in addition, CU traits were linked to distinct hyper-connectivity to frontal, parietal, and cingulate areas. On this subject, Short et al. [107] showed that a dysfunctional amygdala role induces emotional biases, i.e., erroneous attributions of the hedonic value of stimuli.

### 3.5. Context

For many years, researchers have investigated family and scholar contexts to explain the presence of CU traits in children or adolescents with BD. Table S5 summarizes 22 studies that have investigated the links between children and adolescents’ CU traits and their social contexts. Such relations have been analyzed using a number of different tools and methods: parents and teachers report questionnaires, interviews, parental observation, and also with hormonal measures. Researchers focused on two specific contexts in order to better understand the bidirectional effects of CU traits: family context (17/22) and, to a lesser extent, school context (5/22). Results focused on family context indicate four important outcomes: (1) longitudinal studies show that emotional specificities appear in early childhood, before two years old, and persist in the absence of intervention, e.g., [87,113]; (2) research points to inconsistent parenting, lack of contingencies, coercive processes, punitive education associated with a lack of educational framework inducing a reversal of the parent-child role, e.g., [114,115]. Parents-child relationships are characterized by mutual anger, emotional maladjustment, lack of contingency between child behaviors and adults’ responses, or on the contrary, by an emotional insensitivity [113]; (3) insecure attachment (in particular maternal attachment), a lack of parental sensitivity or monitoring are correlated to high CU traits, e.g., [94,116]. Consistent with this, Bedford et al. [117] found that the interaction between infants’ mother-directed gaze and maternal sensitivity played significant roles in the development of later CU traits; (4) the poor responsiveness of these children to punishment exposes them to a high risk of developing particularly severe and stable antisocial behavior [118]; and (5) longitudinal studies demonstrate the efficiency of parent training, e.g., [87,106], to decrease child BD and CU traits [119]. For this purpose, the study conducted by Kimonis [87] revealed that parent-child interaction intervention has multiple benefits. Their outcomes underline that three months posttreatment, 75% of treatment completes no longer showed clinically significant CD relative to 25% of dropouts. Findings of Kochanska [120] indicate indirect effects of family training on youth attitude toward substance use.

Results focused on school context indicate three important outcomes: (1) teachers are essential actors of socialization, emotional regulation and are important attachment figures for children and adolescents [4,70]; (2) longitudinal studies found that children with CU traits developed the highest levels of conflict with teachers and the lowest degree of closeness to them [70]. Youth with CU traits demonstrated more conflicted relationships with their teachers, characterized by displays of anger [4,119]; and (3) this incapacity to develop positive relationships with their authority figures is linked to their disruptive behaviors, emotional dysregulation, and inhibition difficulties. As mentioned by Horan [119], mismatches between children’s needs, teacher expectations, and discipline approaches make positive student-teacher relationships difficult to develop and maintain.

### 3.6. Social Adjustment

For many years, researchers have been investigating the bidirectional effects of CU traits on the child’s social adjustment. Whether measured by parents or by children or adolescents themselves, CU traits tend to remain stable from childhood to adolescence and predict antisocial behavior in adulthood [109]. Table S6 summarizes eight studies that investigated the links between children and adolescents with CU traits and their social adjustment. Such relations have been analyzed using a number of different tools and methods such as hormonal measures, questionnaires, interviews, parental observation, heart rate. Results focused on this dimension indicate four important outcomes: (1) results of all studies (8/8) provide evidence that early life displays of CU traits often persist and are linked with aggressive behavior [77,94]; (2) correlations with prior trauma exposure influence social adjustment and the antisocial trajectory [121]; (3) developmental heterogeneity is explicated by the levels of social competence [80] and the capacity of self-regulation [109]. Emotional overmodulation is a factor associated with recidivism of delinquency [111]; and (4) the role of positive parenting as a protective factor is emphasized [120].

### 3.7. Social Interactions and Social Roles

For many years, researchers have been investigating how CU traits influence the social interactions in populations with BD. Table S7 summarizes 10 studies that investigated the effects of CU traits on children and adolescent social interactions and roles. Such relations have been found using a number of different tools and methods such as interviews, questionnaires, etc. Results indicate three important outcomes: (1) these traits tend to be stable from childhood to adolescence or adulthood, and significant links between CU traits and maladaptation and aggression were found by most of the studies (10/10), e.g., [79,111]; (2) change is possible with multimodal interventions, including in the youth environment [87]; and (3) the negative impact of CU traits reaches beyond a youth family, social interactions and ultimately affecting society as a whole (e.g., delinquency, school dropouts, substance addiction, or difficulties in work life) [122].

### 3.8. Cognition

For many years, researchers have been investigating CU traits and attentional, emotional processes in populations with BD. Table S8 summarizes 19 studies investigating the place and role of neurocognitive mechanisms linked to CU traits. Such relations have been found using a number of different tools and methods (self-reported questionnaires, observations, interviews, standardized tools, fRMI, etc.). Results indicate five important outcomes: (1) he most consistent findings are that two cognitive processes, independent but complementary, are associated with CU traits in children and adolescents: on the one hand, deficits of executive functions, on the other hand, negative school performances: (1) all the studies (12/19) measuring the EF found that children with high levels of CU traits have significantly poorer executive functions [104]. These deficits significantly impact their emotional recognition, their emotional regulation, and self-regulation compared to groups without CU traits [41,78]; (2) the authors underline that these features have severe consequences on their understanding and interpretation of cognitive or social situations [123], their ability to inhibit automatic responses, to adopt reflective skills before acting, to find adjusted solutions to their social context; (3) as mentioned by Aghajani et al. [69], results of fRMI show disturbance in neural correlate that perturb attention-emotion-interactions in children and adolescents with CU traits and impact salience processing and associative learning. Authors underline the over effortful control in these children, which is a self-regulatory capacity to inhibit a dominant response and/or to activate a subdominant response [106]. These outcomes are in line with the work of Withe et al. [112], which highlighted that ventromedial prefrontal cortex responsiveness and ventromedial prefrontal cortex-amygdala connectivity are related to behavioral responses and reactive aggression; (4) the great deficits in executive functioning are a way to explicate pronounced their levels of thrill-seeking and adventurous behavior [109]; and (5) while links to IQ level have not been proven [78,81], the research found links between CU traits and students’ academic performance. Results of Bird et al. [7] reveal that CU traits are significantly related to lower English, Math, and Science grades (lower Science grades for boys only, not for girls). Similarly, Crum et al. [70] found that CU traits are robustly associated with a functional deficit at the end of the school year (p. 3).

## 4. Discussion

The purpose of this systematic review was to examine studies showing bidirectional effects of CU traits with each component of human functioning. Results are first discussed in relation to the characteristics of the samples included in our review and to the tools used for measuring CU traits, and then in regard to the five components of human functioning. Thereafter, reflections on strengths and limitations are mentioned, then recommendations for practice and future research are proposed.

### 4.1. Characteristics of Samples

The ratio of girls to boys present in the studies is consistent with the prevalence of CD by sex. Previous research has demonstrated an important sex difference in the various categories of BD [124]. Research reports a prevalence of ODD in childhood at around 3–4%, higher in boys (4–5%) than in girls (2–3%) and in adolescence, at around 1–3%, also higher in boys (2–4%) than in girls (1–2%) [1,86]. Only a few studies included in the review have examined the influence of sex on CU traits, and none have been adequately powered to test whether the relationship between CD and structural connectivity differs by sex [86]. These observations highlight the importance of considering sex differences when studying the neurobiological basis of youth with CD. Concerning the mean age targeted by the studies, data are consistent with those from the literature, with a peak of the prevalence of ODD in childhood for both sexes (around 8–10 years) and then a decline. The prevalence of CD increases up at around 14–16 years, and thereafter, it remains stable in boys while it decreases in girls [114]. In line with previous research, e.g., [18], our outcomes emphasize the need to appreciate sex differences in biological, psychological, and social factors associated with puberty, such as hormonal changes, decreased parental supervision, and peer group affiliation.

### 4.2. Measure of CU Traits

In the reviewed studies, CU traits are mostly assessed by the ICU rating scale and by a single informant, mostly parent/caregiver and the child/youth himself (see also Table S2). Since dishonesty and deviant insight are among the essential characteristics of psychopathy features, the reliability and validity of self-assessments require careful evaluation [79]. While parents are a very important source of information, they tend to identify only the most severe forms, especially in families with several children with BD [16,125]. In addition, teachers are not often required to assess CU traits, and few studies simultaneously used two (9/52) or more (n = 0) sources. Yet, the prevalence of BD could be underestimated without the advice of teachers who are very sensitive sources of information, even if they tend to overestimate the symptoms when the cultural distance with the child is great [114]. While self-report inventories are increasingly used in the assessment of youth psychopathology because they are relatively easy to complete [78], it is important to use them with caution, as these assessments are de facto subjective. Indeed, social desirability can have a strong influence on the scores of the three sub-dimensions of the ICU [126]. Self-reporting scales raise legitimate questions about the truthfulness of reports by young people who tend to flout the truth and/or violate social rules.

Despite they are time-consuming, interview-based tools as the Psychopathy Checklist: Youth Version (PCL: YV [127]) or the Clinical Assessment of Prosocial Emotion (CAPE; [128]) are also of interest to gain richer information and help, for the latter, making diagnosis decisions based on the DSM-5 LPE specifier criteria [66,128]. In addition, the Child Problematic Traits Inventory (CPTI; [129]) could be of interest when examining CU traits and other correlate features (i.e., grandiose, deceitful interpersonal style, impulsivity, need for stimulation) in young children (3–12 years old) as well with adolescents [130]. This scale is also of interest because it has been shown to be able to predict violent and antisocial behaviors in youth [129,131]. In addition, unlike the PCL: YV, the CPTI, as other scales such as the ICU and the YPI, is useful not only with institutionalized or incarcerated youth but also with community samples or non-referred youths.

### 4.3. Mental Health Dimension

As evidenced by the high numbers of studies results in this dimension, better understanding children’s mental health with high CU traits is a major research challenge. Researchers’ work has focused largely on comorbidities (internalized and/or externalized disorders, neurodevelopmental disorders such as ADHD, etc.), personality traits (lack of remorse, deficient affect, lack of empathy), and emotional regulation disorders. Although CU traits have been more investigated in relation to externalizing behavior (30/36 studies), our results show links between CU traits and internalizing problems, even if their association seems less straightforward [16,132]. Contradictory results for internalized disorders may probably be explained by the difficulty in assessing internalized disorders and also by the unreliability of self-report questionnaires by children, as mentioned by the literature [79]. Despite youth with CU traits have often been described as displaying lower levels of neuroticism (i.e., lower levels of anxiety and depression [53], our results show comorbidity between CU traits and internalized disorders as anxiety and depression. This is consistent with the results of the review by Colins et al. [66], which found no group differences between youth with conduct disorder and the “Limited Prosocial Emotions” specifier and youth with CD only.

In line with previous research [11,114], outcomes show that CD is rarely isolated and presents strong comorbidities with other disorders. Above all, these results highlight the importance of early differential diagnosis to better understand the developmental implications of CU traits, taking into account internalizing disorders but also other neurodevelopmental disorders such as ADHD and ASD. Another important finding is that CU traits are not specific to children with CD but may also be observed in children with other disorders as ASDs or ADHD. This outcome combines with other results fields as neuroscience [28], also supports Raine [34] and Wakshlag et al. [61] conclusion that early disruptive behaviors could be neurodevelopmental in nature. Thus, its classification as an early childhood neurodevelopmental state in the DSM-5 should be discussed. Indeed, Reidy et al. [28] support the idea that there is a neurodevelopmental basis for the core interpersonal-affective dysfunction in psychopathy (see also [133]). Several other indications presented in our review also support the growing hypothesis that not only early disruptive behavior but also antisocial personality disorder have a neurodevelopmental origin [134]: origins in (early) childhood, stability throughout childhood and adolescence into adulthood, higher rates in male, comorbidity with other neurodevelopmental disorders (i.e., ADHD), brain abnormalities, heritability, early life stressors, abuse, trauma and low parental warmth leading to emotional dysregulation and aggression, neurocognitive impairments as poor executive functions, poor facial recognition, and reward dominance [134]. These indicators studied and discussed in the present review for CU traits in youth are also a precursor to the development of APD [134].

Another aspect related to the mental health dimension concerns the importance of emotional disorders in young people with high CU traits. Affecting approximately one-third of youth with BD [70], CU traits are associated with significant dysfunctions in emotion recognition, manifested in particular by deficient responses to fear and a low tolerance level to frustration, corroborating results of previous literature reviews, e.g., [24,33,53]. Outcomes, in line with previous results’ studies, e.g., [24,53] underline that emotions are the main feature of all human behavior, including socialization, social interactions, cognition, social adjustment, and empathy favoring the ability to recognize other people’s emotions, the ability to assume the point of view of others, and the ability to show sympathy or sensitivity to others [114,135,136]. Thus, studies provided evidence that young with high CU traits process emotional stimuli differently from those with CD and low CU traits [137]. Lastly, neuroscience perspectives on behavior disorders have begun to investigate how neurobiological processes influence the relationship between early-life exposure to social adversity and persistent behavioral deficits bringing new insights into this field of study [84].

### 4.4. Neurophysiological Health

Neuroanatomical and functional variations in youths with CD have been reported with increased frequency since the advent of modern neuroimaging [122]. If previous research examined the amygdala as a unitary structure, recent studies examined how amygdala subregional network function affects the features of CU traits in children with BD [69]. Hence, little is yet known about how amygdala subregional network function contributes to CU traits among the diversity of comorbidity linked to BD. Our outcomes underline that limbic structures play a key role in the control of emotions, while self-control, motivation, and acting-out involve the anterior cingulate and orbitofrontal cortex. It is precisely in these regions that changes in the activity of neural systems obviously play a role in the expression and control of these behaviors [114]. Although research in modern neuroimaging makes valuable contributions in the field, it is also necessary to mention its limits. Actually, no study on neuroimaging measurements is longitudinal, failing to show how specific processing biases develop or evolve [25,26]. For this purpose, Viding and McCrory [26] recommend that future neurocognitive functioning data are assessed within other valuable environmental data (e.g., risk and protective factors, epigenetic data). In addition, researchers selected their samples on the basis of self-reported or hetero-reported questionnaires. Results should therefore be taken with caution, especially since Levy’s research found significant links between CU traits and oxytocin level only from the results of the ICU-TR and not from the ICU-SR [79]. Moreover, studies are mostly correlational and cannot claim causality [34,60]. Lastly, studies using neuroimaging have, in general, a small sample size (e.g., [69,83,112]) due to possible methodological and/or financial restrictions, which may result in studies being sufficiently powerful to detect significant effects, as underlined by previous research, e.g., [34]. Despite these limitations, more neuroimaging studies are needed, especially by comparing children and adolescents with comorbid disorders (CD, ODD, ADHD, ASD) with and without high CU traits, as well as studies taking sex into account to study structural brain differences between male and female [33]. These studies are promising for potentially clarifying the neurological markers for some of the emotional and cognitive characteristics of children and adolescents with severe BD and high CU traits.

### 4.5. Context

With regard to the context dimension, results of the review have shown that the family context is a key factor both in the development of BD and in the stability of CU traits [8]. The family environment could have a protective or aggravating role in the social adjustment of youth with CU traits. Many studies mention the risks in the family context of BD children, especially if the parents present antisocial patterns, criminal backgrounds, or addictive behaviors. While previous research has shown that children with high CU traits are particularly resistant to behavioral and family-based treatments, our results tend to contradict this assertion [87], as several longitudinal studies report the efficiency of interventions focusing on children and their families, e.g., [106,119]. These inconsistent results can probably be explained by several factors such as the setting of very early, longitudinal, and multimodal interventions in these families (some with more than six measurements times) and the inclusion of both child and parent in treatment. Despite youth with CU traits would respond to parenting/family interventions, Viding and McCrory [26] (p. 573) note that biological parents of a CU child may themselves share some of their child’s vulnerabilities and may find it more difficult to implement elements of typical parenting intervention programs (e.g., parental warmth, positive reinforcement). Biological families of children with CU traits may require additional clinical support (or different forms of support) to facilitate the development and implementation of more positive models of parent-child interactions [138].

Results of the review not only highlight the importance of the relationship with parents but also with teachers [4] and peers [109], although little or no research was conducted in the school context. Overall, outcomes point out teachers’ role as models and emotional regulators for youth with CU traits [4]. Impaired relationships between children with CU traits and their teachers are particularly worried because teachers are key attachment figures for children, and the quality of their relationship could lead to feelings of security and positive adaptive behaviors [70]. Better teacher knowledge of CU traits could be particularly important in influencing the developmental trajectory of these youths. For example, if prevention strategies (e.g., positive reinforcement of prosocial behaviors) in school are of interest, teachers must know that children and adolescents with high CU traits are less sensitive to social reinforcers. Therefore, they have first to identify, for each young people, the subjective reward likely to reinforce their prosocial behaviors [28,139]. In addition, according to Lui et al. [9], most programs led by trained teachers, in group settings and targeting the development of perspective-taking skills show positive outcomes, especially if they integrate different components (role-playing including those derived from participants’ scenarios, discussions, modeling, rehearsal), in addition to a certain length (6 to 10 weeks).

Our outcomes also underline the importance of considering multiple environmental determinants in examining the CU traits, suggesting the need to integrate and expand previous research beyond the family context to include the child’s other life environments, particularly the school context. As already noted by Crum, no research has yet examined the effects of a school-based intervention on students’ CU traits [70], a promising but under-explored area of research.

### 4.6. Cognition

Research results in neurophysiology, neuroscience, and neuroanatomical imaging demonstrate the links between executive functions and the correct functioning of the frontal lobes [114]. Aligned with brain dysfunction’s models, our review outcomes underline the over effortful control necessary for children with high CU traits to inhibit a dominant response and/or to activate a subdominant response due to their self-regulatory deficits [2,106]. Our findings are consistent with previous research and/or reviews that documented significant deficits in executive function among youth with CU traits and CD [24,25,35]. In addition, in line with Frick’s work [24], outcomes point out various deficits in executive functions, emotional regulation, inhibition that impact several core functioning dimensions of their cognitive abilities. Indeed, executive functions cover all the processes necessary for the achievement of complex and goal-oriented tasks: analysis of external information, clarity of purpose, elaboration and planning of response, anticipation of consequences, consideration of changing environmental information and inhibition of irrelevant responses, conscious control of its execution [2]. Any disturbance of executive functions hinders the ability to act in a controlled manner, by increasing impulsive and inappropriate responses in social contexts [43]. Thus, children with CU traits tend to experience school difficulties, lack of self-esteem, lack of motivation at school, lack of concern for school performances, school absenteeism, and school dropout [18]. These findings of impulsivity and indifference to the consequences of the actions taken corroborate the more actual thesis of a multidimensional conceptualization of psychopathic features in children and adolescents (i.e., daring-impulsive, behavioral dimension), contradicting here the one-dimensional theoretical model reducing these traits to CU only (i.e., affective dimension) [130,140]. The third dimension of this model, the grandiose-manipulative dimension, is relative to interpersonal functioning (e.g., lying, manipulation, domination of others) detailed below.

### 4.7. Social Interactions (and Social Roles)

Concerning the social interactions dimension, longitudinal studies included in our review seem to confirm that early childhood CU traits are associated with the severity of conduct problems in adolescence and with antisocial trajectory into adulthood [141] in the absence of multimodal intervention. The transition from childhood to adolescence is recognized as a particularly sensitive lifetime, as it solicits numerous relevant coping skills [120]. In accordance with previous work, there is some evidence that CU traits have an impact on the social roles of children or young people [134]. Youth with CU traits were found to have lower levels of social adjustment and to incorporate deviant peer groups [105]: difficulties in engaging prosocial behaviors, both proactive and reactive forms of aggression, difficulties in cooperating, risky and delinquent behaviors [4] substance abuse [69]. Propagation of deviant behaviors is reinforced by belonging to antisocial peer groups, which is a counter-cultural attitude that promotes aggressive behavior and transgressions of societal rules [84]. Children with CU traits are likely to seek out peers who are similar to them and to adopt values that contradict the socialization messages of their home environment [120]. In accordance with Zuckerman’s psychobiological model, their search for strong sensations is associated with a weak activation of the catecholaminergic system, particularly dopaminergic [84]. Outcomes show that in a non-stimulation situation, children seek a catecholaminergic increase through substance abuse or risky behavior. In line with neurobiological models, children with CU traits exhibited increased biological vulnerability to emotional processing as evidence by hypo-arousal within the defensive responsive system. Together, these abnormalities may contribute to antisocial behavior and deviant peer group affiliation [105].

### 4.8. Social Adjustment

In addition to genetic predispositions, the influence of the social environment should be considered as an additional factor in explaining severe conduct disorders [16]. According to Fanti’s work, genetic studies’ results included in our review have demonstrated the shared and unshared environmental influences in the predisposition of individuals to antisocial behavior. These results, in line with well-established literature [6,10], show that the presence of CU traits during early childhood is a strong developmental risk factor for antisocial and aggressive behavior persisting throughout childhood and adolescence [105]. The progression of antisocial behavior is shaped by antisocial attitudes, poor schooling, and a lack of affiliation with prosocial peers [84]. Youth with CU traits are thrill-seeking, find benefit in their antisocial behavior, and appear to be unconcerned about the negative consequences of their behaviors [121], and these factors negatively impact their social adjustment.

### 4.9. Implications for Practice

Our research has several implications in terms of both prevention and intervention. Since CU traits emerge in early childhood and often persist into childhood and adolescence, preventive interventions are needed to identify them at a very early stage [84]. Common intervention is known to produce inconclusive results with youth with antisocial behaviors, CD, and high CU traits [24], as well as with antisocial personality disorders (APD) adults [134]. Considering early disruptive disorders, CU traits and APD in a neurodevelopmental approach could allow a change of perspective and advocate a need for early interventions [134]. It is thus worthy of considering early health interventions to prevent brain structure abnormalities and impairments in brain functions, with nutrient supplementation in pregnancy and/or childhood (e.g., omega 3, zinc) as well as early environmental enrichment (better nutrition, physical exercise, cognitive stimulation, sleep hygiene) [134,142]. Raine et al. [142] have indeed demonstrated the effects of omega-3 dietary supplementation on the long-term reduction of behavioral problems in children. A significant decrease in aggressive and antisocial behavior of the parents of the supplemented children was also found. This improvement in parental behavior is partly responsible for the observed improvements in children’s behavior [142], again highlighting the role of person-by-context interactions in the development of CU traits.

At the family and (pre)school context level, programs that target a reduction in sanctions in favor of positive reinforcement of prosocial behaviors show promising results not only in terms of reducing acts of violence but also in terms of reducing CU traits [28,65]. Our review results seem to indicate that CU traits can improve with multilevel and multimodal treatments [6]. It has been demonstrated that both brain structure and function may be modified by experience [74]. Activation-dependant structural plasticity can even occur after about seven days of training [122], and it is suggested to play a key role in human adaptation to environmental changes and disease. Nevertheless, Wilkinson et al. [56] call for more research in this area to evaluate the effectiveness of interventions, especially those that target areas of functioning that are particularly deficient in youth with CU traits, such as programs focusing on emotion recognition (see also [57,58]), but also programs that focus on CU behaviors in early childhood (e.g., parenting interventions [143]). Finally, the intervention should take into account the influence of the level of severity of CU traits on the response to the intervention (e.g., [33] for a review).

Consequently, any intervention in the field of BD should not overshadow the evaluation of CU traits or target a single dimension for effective intervention. Measuring CU traits is also valuable for predicting different forms of recidivism [144]. Assessment should therefore be broad enough to take into account that CU traits may be a specification of other disorders (ASD, ADHD) as well as a developmental pathway to severe conduct problems or antisocial behaviors, without systematically meeting full criteria for CD [24,53,145]. In this regard, our results are consistent with the comprehensive reviews conducted by Frick et al. [24,53], providing insight into the use of CU traits to identify a subgroup of children and adolescents with severe conduct problems, both for diagnostic and clinical goals. On this topic, and as suggested by many authors, physicians, early childhood educators, and teachers have a key role in detecting children with CU traits, in supporting their emotional regulation, and in encouraging their social adjustment and social integration [4].

### 4.10. Strengths and Limitations

Our review has several important strengths, as well as limitations, that should be highlighted. In terms of strengths, the present study has a fairly large number of studies that enabled us to systematically investigate the impact of CU traits in the different functioning dimensions of children and adolescents with BD. Our outcomes, in line with previous work, are important both from an etiological and developmental point of view because they underline the necessity of the very early identification of emotional particularities that tend to remain stable in time without early detection and intervention [114]. Furthermore, the AAIDD’s model provides a rich and scientifically valid theoretical framework for identifying the various biological, psychological, cognitive, social, and contextual dimensions of youth while focusing attention on the supports required to improve individual functioning in young people with severe BD. Findings seem to demonstrate the interest of having adopted a model in order to better understand the multidirectional effects of high CU traits in children and adolescents.

Despite these strengths, our review also has limitations. A potential limitation concerns the overall sample is significantly lower in girls than in boys without differentiating the results by sex. Common to many studies, CU traits were assessed in male samples, with wide age ranges, without systematic clinical levels of BD, which make it difficult to draw strong conclusions about the nature of associations between CU traits and BD [113]. Furthermore, it remains unclear whether identified brain alterations in boys with BD are also present in girls with BD. In addition, unfortunately, only limited work investigated the interactive effects between ASD, ADHD, or other neurodevelopmental disorders and CU traits in predicting empathy deficits [101]. Another limitation refers to the assessment of CU traits, carried out mostly by a single informant and often using a self-reported questionnaire [79]. Furthermore, we choose to report results concerning youth with high CU only, so our review does not provide an overview of the effects for youth with low CU traits. However, this choice was especially guided by the fact that young people with high CU traits are known to form a subgroup of young individuals with the most severe and violent forms of conduct disorder [144]. Moreover, inclusion criteria focused on the affective dimension of psychopathic features in youth (i.e., CU traits), limiting our findings and conclusions to a broader conceptualization of pre-psychopathic traits (i.e., tridimensional model, Garcia et al. [31]). A final limitation is related to the model itself: while it facilitated the report of the effects of CU traits across the different dimensions of human functioning, the categorizations of outcomes across the different dimensions may be debatable. In particular, the social adaptation dimension could have included all the studies. Selections made could appear reductive, even if they were intended to provide a better insight into how these young people are functioning in their daily lives.

### 4.11. Further Research

Future research should ensure that CU traits are evaluated with multiple informants, including teachers, in order to refine the understanding of CU traits, distinguishing their effects when related to internalized and/or externalized disorders. It should also consider more targeted treatment approaches for each youth affected. In addition, research should incorporate multilevel models for understanding the effects of CU traits on youth health, by differentiating outcomes by age and sex, as hormonal factors—and their disruption in adolescence – cannot and should not be neglected. Further research should also focus on specific hormonal factors, differentiating results by sex, by age, and by behavioral disorders. As recommended by Fragkaki et al. [146], research on the hormonal activity should take into account different risk factors leading to aggression, such as trauma experience, as it seems to be strongly correlated with CU traits pathways, e.g., [121,134], possibly by affecting the hormonal interplay of cortisol, testosterone, and oxytocin in adolescent aggression.

To date, little research has been conducted in the school setting [4], particularly in the French-speaking community, to assess the presence of CU traits in populations at risk. Given the impact of these traits on youth functioning, this is an important gap to be covered by future research. Due to the lack of research including French-speaking samples (see also Garcia et al., [13]), we also encourage further research assessing CU traits in French samples, in particular by the French validation of questionnaire used to measure these traits.

## 5. Conclusions

The purpose of this review was to offer an overall overview of how CU traits influence the dimensions of children and adolescent functioning. Outcomes point out that youth with CU traits are a heterogeneous group with multiple disorders in their functional abilities due essentially to emotional dysregulation. More specifically, outcomes indicate aberrant brain functioning in the main areas of the brain, specifically disturbances in the prefrontal cortex (orbitofrontal, dorsolateral and medial), limbic (e.g., amygdala, anterior insula, cingulate cortex), and temporal cortex. Disturbances in these areas impact both emotional processing and regulation, executive functions [143], decision-making [147], empathy, passive avoidance, etc. Considering the effects of these traits on the youth functioning, an intervention focused on the presence of CU traits is a necessary but not a sufficient approach. More specific knowledge of the effect of CU traits on the psycho-developmental profile of children and adolescents is required to adjust each individualized intervention plan. This study also highlights the need for more longitudinal research on early childhood intervention programs to examine their effects on later development. Such research is needed because these traits are associated with severe and often stable antisocial behaviors over time [148]. Finally, and because of the real limitations in the field, we agree with Colins et al. (2020) that the DSM-5 LPE specifier should be used with caution for diagnostic and treatment planning purposes. As the CU traits are not pathognomonic of violence [28], nor are they immutable [149], we must consider that young people with serious behavioral problems with or without CU traits are still developing and capable of evolving in different equally positive ways.

## Data Availability

Data are available on request.

## Supplementary Materials

The following are available online at https://www.mdpi.com/article/10.3390/ijerph18094712/s1: Table S1. Characteristics of the studies included in the review; Table S2. Summary of studies design, key methods, and principal instruments; Table S3. Summary of results—mental health; Table S4. Summary of results—neurobiological markers; Table S5. Summary of results—family and school context; Table S6. Summary of results—social adjustment; Table S7. Summary of results—social interactions and social roles dimension; Table S8. Summary of results—cognitive dimension. References [7,21,36,69,70,71,72,73,74,75,76,77,78,79,80,81,82,83,84,85,86,87,88,94,96,99,101,104,105,106,107,108,109,110,111,112,113,114,115,116,117,118,119,120,121,122,123,124,125,126,127,128] are cited in the Supplementary Materials.

## References

[1] (2013). American Psychiatric Association Diagnostic and Statistical Manual of Mental Disorders.

[2] Frick P.J. (2012). Developmental Pathways to Conduct Disorder: Implications for Future Directions in Research, Assessment, and Treatment. J. Clin. Child Adolesc. Psychol..

[3] Piquero A.R., Shepherd I., Shepherd J.P., Farrington D.P. (2011). Impact of offending trajectories on health: Disability, hospitalisation and death in middle-aged men in the Cambridge Study in Delinquent Development. Crim. Behav. Ment. Health.

[4] Baroncelli A., Ciucci E. (2020). Bidirectional Effects Between Callous-Unemotional Traits and Student-Teacher Relationship Quality Among Middle School Students. J. Abnorm. Child Psychol..

[5] Waschbusch D.A., Walsh T.M., Andrade B.F., King S., Carrey N.J. (2007). Social problem solving, conduct problems, and callous-unemotional traits in children. Child Psychiatry Hum. Dev..

[6] Frick P.J., Ray J.V. (2015). Evaluating Callous-Unemotional Traits as a Personality Construct. J. Pers..

[7] Bird E., Chhoa C.Y., Midouhas E., Allen J.L. (2019). Callous-Unemotional Traits and Academic Performance in Secondary School Students: Examining the Moderating Effect of Gender. J. Abnorm. Child Psychol..

[8] Ciucci E., Baroncelli A. (2014). Emotion-Related Personality Traits and Peer Social Standing: Unique and Interactive Effects in Cyberbullying Behaviors. Cyberpsychol. Behav. Soc. Netw..

[9] Lui J.H.L., Barry C.T., Sergiou C.S. (2017). Interventions for Improving Affective Abilities in Adolescents: An Integrative Review Across Community and Clinical Samples of Adolescents. Adolesc. Res. Rev..

[10] Frick P.J., White S.F. (2008). Research Review: The importance of callous-unemotional traits for developmental models of aggressive and antisocial behavior. J. Child Psychol. Psychiatry Allied Discip..

[11] Frick P.J., Stickle T.R., Dandreaux D.M., Farrell J.M., Kimonis E.R. (2005). Callous-unemotional traits in predicting the severity and stability of conduct problems and delinquency. J. Abnorm. Child Psychol..

[12] Frick P.J., Kimonis E.R., Dandreaux D.M., Farell J.M. (2003). The 4 Year Stability of Psychopathic Traits in Non-Referred Youth. Behav. Sci. Law.

[13] Garcia M., Rouchy E., Michel G. (2019). The role of Callous-Unemotional traits in delinquent and criminal trajectories: A review of longitudinal studies. Ann. Med. Psychol..

[14] Frick P.J. (2004). The Inventory of Callous–Unemotional Trait.

[15] Cardinale E.M., Marsh A.A. (2020). The Reliability and Validity of the Inventory of Callous Unemotional Traits: A Meta-Analytic Review. Assessment.

[16] Kimonis E.R., Frick P.J., Skeem J.L., Marsee M.A., Cruise K., Munoz L.C., Aucoin K.J., Morris A.S. (2008). Assessing callous–unemotional traits in adolescent offenders: Validation of the Inventory of Callous–Unemotional Traits. Int. J. Law Psychiatry.

[17] Essau C.A., Sasagawa S., Frick P.J. (2006). Callous-unemotional traits in a community sample of adolescents. Assessment.

[18] Ciucci E., Baroncelli A., Franchi M., Golmaryami F.N., Frick P.J. (2014). The association between callous-unemotional traits and behavioral and academic adjustment in children: Further validation of the inventory of callous-unemotional traits. J. Psychopathol. Behav. Assess..

[19] Roose A., Bijttebier P., Decoene S., Claes L., Frick P.J. (2010). Assessing the affective features of psychopathy in adolescence: A further validation of the inventory of callous and unemotional traits. Assessment.

[20] Pechorro P., Ray J.V., Barroso R., Maroco J., Gonçalves R.A. (2016). Validation of the Inventory of Callous-Unemotional Traits Among a Portuguese Sample of Detained Juvenile Offenders. Int. J. Offender Ther. Comp. Criminol..

[21] Ezpeleta L., Granero R., de la Osa N., Domènech J.M. (2017). Developmental trajectories of callous-unemotional traits, anxiety and oppositionality in 3–7 year-old children in the general population. Pers. Individ. Differ..

[22] Byrne B.M. (2016). Structural Equation Modeling with AMOS: Basic Concepts, Applications and Programming.

[23] Ray J.V., Frick P.J. (2020). Assessing Callous-Unemotional Traits Using the Total Score from the Inventory of Callous-Unemotional Traits: A Meta-Analysis. J. Clin. Child Adolesc. Psychol..

[24] Frick P.J., Ray J.V., Thornton L.C., Kahn R.E. (2014). Can callous-unemotional traits enhance the understanding, diagnosis, and treatment of serious conduct problems in children and adolescents? A comprehensive review. Psychol. Bull..

[25] Umbach R., Berryessa C.M., Raine A. (2015). Brain imaging research on psychopathy: Implications for punishment, prediction, and treatment in youth and adults. J. Crim. Justice.

[26] Viding E., McCrory E.J. (2018). Understanding the development of psychopathy: Progress and challenges. Psychol. Med..

[27] Macrì S., Zoratto F., Chiarotti F., Laviola G. (2018). Can laboratory animals violate behavioural norms? Towards a preclinical model of conduct disorder. Neurosci. Biobehav. Rev..

[28] Reidy D.E., Krusemark E., Kosson D.S., Kearns M.C., Smith-Darden J., Kiehl K.A. (2017). The Development of Severe and Chronic Violence Among Youth: The Role of Psychopathic Traits and Reward Processing. Child Psychiatry Hum. Dev..

[29] Waller R., Hyde L.W. (2017). Callous–Unemotional Behaviors in Early Childhood: Measurement, Meaning, and the Influence of Parenting. Child Dev. Perspect..

[30] Moore A.A., Blair R.J., Hettema J.M., Roberson-Nay R. (2019). The genetic underpinnings of callous-unemotional traits: A systematic research review. Neurosci. Biobehav. Rev..

[31] Salekin R.T. (2017). Research Review: What do we know about psychopathic traits in children?. J. Child Psychol. Psychiatry Allied Discip..

[32] Waller R., Hyde L.W. (2018). Callous-unemotional behaviors in early childhood: The development of empathy and prosociality gone awry. Curr. Opin. Psychol..

[33] Baker R.H., Clanton R.L., Rogers J.C., De Brito S.A. (2015). Neuroimaging findings in disruptive behavior disorders. CNS Spectr..

[34] Ling S., Raine A. (2018). The neuroscience of psychopathy and forensic implications. Psychol. Crime Law.

[35] Herpers P.C.M., Scheepers F.E., Bons D.M.A., Buitelaar J.K., Rommelse N.N.J. (2014). The cognitive and neural correlates of psychopathy and especially callous-unemotional traits in youths: A systematic review of the evidence. Dev. Psychopathol..

[36] Waller R., Hyde L.W., Klump K.L., Burt S.A. (2018). Parenting Is an Environmental Predictor of Callous-Unemotional Traits and Aggression: A Monozygotic Twin Differences Study. J. Am. Acad. Child Adolesc. Psychiatry.

[37] Waller R., Gardner F., Hyde L.W. (2013). What are the associations between parenting, callous–unemotional traits, and antisocial behavior in youth? A systematic review of evidence. Clin. Psychol. Rev..

[38] Hyde L.W., Waller R., Trentacosta C.J., Shaw D.S., Neiderhiser J.M., Ganiban J.M., Reiss D., Leve L.D. (2016). Heritable and nonheritable pathways to early callous-unemotional behaviors. Am. J. Psychiatry.

[39] Waller R., Trentacosta C.J., Shaw D.S., Neiderhiser J.M., Ganiban J.M., Reiss D., Leve L.D., Hyde L.W. (2016). Heritable temperament pathways to early callous-unemotional behaviour. Br. J. Psychiatry.

[40] Kam C.-M., Greenberg M.T., Walls C.T. (2003). Examining the role of implementation quality in school-based. Prev. Sci..

[41] Graziano P.A., Landis T., Maharaj A., Ros-Demarize R., Hart K.C., Garcia A. (2019). Differentiating Preschool Children with Conduct Problems and Callous-Unemotional Behaviors through Emotion Regulation and Executive Functioning. J. Clin. Child Adolesc. Psychol..

[42] Curtis A., Harries T., Moulds L., Miller P. (2019). Addressing child-to-parent violence: Developmental and intervention considerations. J. Fam. Stud..

[43] Kuay H.S., Tiffin P.A., Boothroyd L.G., Towl G.J., Centifanti L.C.M. (2017). A New Trait-Based Model of Child-to-Parent Aggression. Adolesc. Res. Rev..

[44] Wang P.W., Hsiao R.C., Chen L.M., Sung Y.H., Hu H.F., Yen C.F. (2019). Associations between callous-unemotional traits and various types of involvement in school bullying among adolescents in Taiwan. J. Formos. Med. Assoc..

[45] Zych I., Ttofi M.M., Farrington D.P. (2019). Empathy and Callous–Unemotional Traits in Different Bullying Roles: A Systematic Review and Meta-Analysis. Trauma Violence Abus..

[46] Kerr M., Van Zalk M., Stattin H. (2012). Psychopathic traits moderate peer influence on adolescent delinquency. J. Child Psychol. Psychiatry Allied Discip..

[47] Mallion J.S., Wood J.L. (2018). Emotional processes and gang membership: A narrative review. Aggress. Violent Behav..

[48] Fanti K.A. (2018). Understanding heterogeneity in conduct disorder: A review of psychophysiological studies. Neurosci. Biobehav. Rev..

[49] Blair R.J.R. (2019). Dysfunctional neurocognition in individuals with clinically significant psychopathic traits. Dialogues Clin. Neurosci..

[50] Waschbusch D.A., Willoughy M.T. (2008). Attention-deficit/hyperactivity disorder and callous-unemotional traits as moderators of conduct problems when examining impairment and aggression in elementary school children. Agress. Behav..

[51] Pisano S., Muratori P., Gorga C., Levantini V., Iuliano R., Catone G., Coppola G., Milone A., Masi G. (2017). Conduct disorders and psychopathy in children and adolescents: Aetiology, clinical presentation and treatment strategies of callous-unemotional traits. Ital. J. Pediatr..

[52] Willoughby M.T., Mills-Koonce W.R., Gottfredson N.C., Wagner N.J. (2014). Measuring callous unemotional behaviors in early childhood: Factor structure and the prediction of stable aggression in middle childhood. J. Psychopathol. Behav. Assess..

[53] Frick P.J., Ray J.V., Thornton L.C., Kahn R.E. (2014). Annual research review: A developmental psychopathology approach to understanding callous-unemotional traits in children and adolescents with serious conduct problems. J. Child Psychol. Psychiatry Allied Discip..

[54] Waller R., Dishion T.J., Shaw D.S., Gardner F., Wilson M.N., Hyde L.W. (2016). Does early childhood callous-unemotional behavior uniquely predict behavior problems or callous-unemotional behavior in late childhood?. Dev. Psychol..

[55] Burke J.D., Waldman I., Lahey B.B. (2010). predictive validity of childhood oppositional defiant disorder and conduct disorder: Implications for the DSM-V. J. Abnorm. Psychol..

[56] Wilkinson S., Waller R., Viding E. (2016). Practitioner Review: Involving young people with callous unemotional traits in treatment-does it work? A systematic review. J. Child Psychol. Psychiatry.

[57] Hawes D.J., Price M.J., Dadds M.R. (2014). Callous-Unemotional Traits and the Treatment of Conduct Problems in Childhood and Adolescence: A Comprehensive Review. Clin. Child Fam. Psychol. Rev..

[58] Dadds M.R., Cauchi A.J., Wimalaweera S., Hawes D.J., Brennan J. (2012). Outcomes, moderators, and mediators of empathic-emotion recognition training for complex conduct problems in childhood. Psychiatry Res..

[59] Masi G., Milone A., Manfredi A., Brovedani P., Pisano S., Muratori P. (2016). Combined pharmacotherapy-multimodal psychotherapy in children with Disruptive Behavior Disorders. Psychiatry Res..

[60] Andrea L.D., Raine A. (2014). Psychopathy: An Introduction to Biological Findings and Their Implications.

[61] Wakschlag L.S., Perlman S.B., Blair R.J., Leibenluft E., Briggs-Gowan M.J., Pine D.S. (2018). The neurodevelopmental basis of early childhood disruptive behavior: Irritable and callous phenotypes as exemplars. Am. J. Psychiatry.

[62] Gajos J.M., Beaver K.M. (2016). The effect of omega-3 fatty acids on aggression: A meta-analysis. Neurosci. Biobehav. Rev..

[63] Liu J., Hanlon A., Ma C., Zhao S.R., Cao S., Compher C. (2014). Low Blood Zinc, Iron, and Other Sociodemographic Factors Associated with Behavior Problems in Preschoolers. Nutrients.

[64] Glenn A.L., Raine A. (2014). Neurocriminology: Implications for the punishment, prediction and prevention of criminal behaviour. Nat. Rev. Neurosci..

[65] Reidy D.E., Kearns M.C., Degue S. (2013). Reducing psychopathic violence: A review of the treatment literature. Aggress. Violent Behav..

[66] Colins O.F., Van Damme L., Hendriks A.M., Georgiou G. (2020). The DSM-5 with Limited Prosocial Emotions Specifier for Conduct Disorder: A Systematic Literature Review. J. Psychopathol. Behav. Assess..

[67] Kochanska G., Boldt L.J., Kim S., Yoon J.E., Philibert R.A., Bell G.R., Crothers L.M., Hughes T.L., Kanyongo G.Y., Kolbert J.B. (2016). What can we learn about emotion by studying psychopathy?. J. Abnorm. Child Psychol..

[68] Schalock R.L., Keith K.D., Verdugo M.Á., Gómez L.E. (2010). Quality of Life Model Development and Use in the Field of Intellectual Disability. Underst. Investig. Response Process. Valid. Res..

[69] Aghajani M., Klapwijk E.T., van der Wee N.J., Veer I.M., Rombouts S.A.R.B., Boon A.E., van Beelen P., Popma A., Vermeiren R.R.J.M., Colins O.F. (2017). Disorganized Amygdala Networks in Conduct-Disordered Juvenile Offenders with Callous-Unemotional Traits. Biol. Psychiatry.

[70] Crum K.I., Waschbusch D.A., Willoughby M.T. (2016). Callous-Unemotional Traits, Behavior Disorders, and the Student–Teacher Relationship in Elementary School Students. J. Emot. Behav. Disord..

[71] Ezpeleta L., Navarro J.B., de la Osa N., Penelo E., Trepat E., Martin V., Domènech J.M. (2017). Attention to emotion through a go/no-go task in children with oppositionality and callous–unemotional traits. Compr. Psychiatry.

[72] Erdogan G.S., Benga O., Marină C. (2017). Attentional orientation patterns toward emotional faces and temperamental correlates of preschool oppositional defiant problems: The moderating role of callous-unemotional traits and anxiety symptoms. Front. Psychol..

[73] Grazioplene R., Tseng W.L., Cimino K., Kalvin C., Ibrahim K., Pelphrey K.A., Sukhodolsky D.G. (2020). Fixel-Based Diffusion Magnetic Resonance Imaging Reveals Novel Associations Between White Matter Microstructure and Childhood Aggressive Behavior. Biol. Psychiatry Cogn. Neurosci. Neuroimag..

[74] Raschle N.M., Menks W.M., Fehlbaum L.V., Steppan M., Smaragdi A., Gonzalez-Madruga K., Rogers J., Clanton R., Kohls G., Martinelli A. (2018). Callous-unemotional traits and brain structure: Sex-specific effects in anterior insula of typically-developing youths. NeuroImage Clin..

[75] Bakker-Huvenaars M.J., Greven C.U., Herpers P., Wiegers E., Jansen A., van der Steen R., van Herwaarden A.E., Baanders A.N., Nijhof K.S., Scheepers F. (2020). Saliva oxytocin, cortisol, and testosterone levels in adolescent boys with autism spectrum disorder, oppositional defiant disorder/conduct disorder and typically developing individuals. Eur. Neuropsychopharmacol..

[76] Wall T.D., Frick P.J., Fanti K.A., Kimonis E.R., Lordos A. (2016). Factors differentiating callous-unemotional children with and without conduct problems. J. Child Psychol. Psychiatry Allied Discip..

[77] Thomson N.D., Gillespie S.M., Centifanti L.C.M. (2020). Callous-unemotional traits and fearlessness: A cardiovascular psychophysiological perspective in two adolescent samples using virtual reality. Dev. Psychopathol..

[78] Winstanley M., Webb R.T., Conti-Ramsden G. (2021). Developmental language disorders and risk of recidivism among young offenders. J. Child Psychol. Psychiatry.

[79] Levy T., Apter A., Djalovski A., Peskin M., Fennig S., Gat-Yablonski G., Bar-Maisels M., Borodkin K., Bloch Y. (2017). The reliability, concurrent validity and association with salivary oxytocin of the self-report version of the Inventory of Callous-Unemotional Traits in adolescents with conduct disorder. Psychiatry Res..

[80] Hitti S.A., Sullivan T.N., McDonald S.E., Farrell A.D. (2019). Longitudinal relations between beliefs supporting aggression and externalizing outcomes: Indirect effects of anger dysregulation and callous-unemotional traits. Aggress. Behav..

[81] Sethi A., O’Nions E., McCrory E., Bird G., Viding E. (2018). An fMRI investigation of empathic processing in boys with conduct problems and varying levels of callous-unemotional traits. NeuroImage Clin..

[82] Rogers J.C., Gonzalez-Madruga K., Kohls G., Baker R.H., Clanton R.L., Pauli R., Birch P., Chowdhury A.I., Kirchner M., Andersson J.L.R. (2019). White Matter Microstructure in Youths With Conduct Disorder: Effects of Sex and Variation in Callous Traits. J. Am. Acad. Child Adolesc. Psychiatry.

[83] Breeden A.L., Cardinale E.M., Lozier L.M., VanMeter J.W., Marsh A.A. (2015). Callous-unemotional traits drive reduced white-matter integrity in youths with conduct problems. Psychol. Med..

[84] Fagan S.E., Zhang W., Gao Y. (2017). Social Adversity and Antisocial Behavior: Mediating Effects of Autonomic Nervous System Activity. J. Abnorm. Child Psychol..

[85] Rizeq J., Toplak M.E., Ledochowski J., Basile A., Andrade B.F. (2020). Callous-Unemotional Traits and Executive Functions are Unique Correlates of Disruptive Behavior in Children. Dev. Neuropsychol..

[86] González-Madruga K., Rogers J., Toschi N., Riccelli R., Smaragdi A., Puzzo I., Clanton R., Andersson J., Baumann S., Kohls G. (2020). White matter microstructure of the extended limbic system in male and female youth with conduct disorder. Psychol. Med..

[87] Kimonis E.R., Fleming G., Briggs N., Brouwer-French L., Frick P.J., Hawes D.J., Bagner D.M., Thomas R., Dadds M. (2019). Parent-Child Interaction Therapy Adapted for Preschoolers with Callous-Unemotional Traits: An Open Trial Pilot Study. J. Clin. Child Adolesc. Psychol..

[88] Takahashi Y., Pease C.R., Pingault J., Viding E. (2021). Genetic and environmental influences on the developmental trajectory of callous-unemotional traits from childhood to adolescence. J. Child Psychol. Psychiatry.

[89] Frick P.J., Hare R.D. (2001). Antisocial Process Screening Device (APSD): Technical Manual.

[90] Andershed H., Hodgins S., Tengström A. (2007). Convergent validity of the youth psychopathic traits inventory (YPI): Association with the psychopathy checklist: Youth version (PCL:YV). Assessment.

[91] Lynam D.R. (1996). Early identification of chronic offenders: Who is the fledgling psychopath?. Psychol. Bull..

[92] Achenbach T.M., Leslie A. (2000). Rescorla Manual for the ASEBA Preschool Forms and Profiles.

[93] Waschbusch D.A., Porter S., Carrey N., Kazmi S.O., Roach K.A., D’Amico D.A. (2004). Investigation of the heterogeneity of disruptive behaviour in elementary-age children. Can. J. Behav. Sci..

[94] Grotzinger O., Grotzinger A.D., Mann F.D., Patterson M.W. (2018). Corrigendum to: Hair and Salivary Testosterone, Hair Cortisol, and Externalizing Behaviors in Adolescents (*Psychol. Sci.*
**2018**, *29*, 688–699, 10.1177/0956797617742981). Psychol. Sci..

[95] Kaufman J., Birmaher B., Brent D., Rao U., Flynn C., Moreci P., Williamson D., Ryan N. (1997). Schedule for affective disorders and schizophrenia for school-age children-present and lifetime version (K-SADS-PL): Initial reliability and validity data. J. Am. Acad. Child Adolesc. Psychiatry.

[96] Masi G., Pisano S., Brovedani P., Maccaferri G., Manfredi A., Milone A., Nocentini A., Polidori L., Ruglioni L., Muratori P. (2018). Trajectories of callous–unemotional traits from childhood to adolescence in referred youth with a disruptive behavior disorder who received intensive multimodal therapy in childhood. Neuropsychiatr. Dis. Treat..

[97] Shi L.J., Ou J.J., Gong J.B., Wang S.H., Zhou Y.Y., Zhu F.R., Liu X.D., Zhao J.P., Luo X.R., Petalas M.A. (2015). To print this document, select the Print. Heal. Psychol. Rep..

[98] Raine A., Dodge K., Loeber R., Gatzke-Kopp L., Lynam D., Reynolds C., Stouthamer-Loeber M., Liu J. (2006). The reactive-proactive aggression questionnaire: Differential correlates of reactive and proactive aggression in adolescent boys. Aggress. Behav..

[99] Craig M.C., Mulder L.M., Zwiers M.P., Sethi A., Hoekstra P.J., Dietrich A., Baumeister S., Aggensteiner P.M., Banaschewski T., Brandeis D. (2019). Distinct associations between fronto-striatal glutamate concentrations and callous-unemotional traits and proactive aggression in disruptive behavior. Cortex.

[100] Constantino J.N., Gruber C.P. (2005). Social Responsiveness Scale.

[101] Georgiou G., Demetriou C.A., Fanti K.A. (2019). Distinct Empathy Profiles in Callous Unemotional and Autistic Traits: Investigating Unique and Interactive Associations with Affective and Cognitive Empathy. J. Abnorm. Child Psychol..

[102] Rutter M., Bailey A., Lord C., Berument S.K. (2003). Social Communication Questionnaire.

[103] Holland D.D.M. (1995). The Diagnostic Interview Schedule for Children, Adolescents, and Parents (DISCAP).

[104] Bedford R., Gliga T., Hendry A., Jones E.J.H., Pasco G., Charman T., Johnson M.H., Pickles A., Baron-Cohen S., Bolton P. (2019). Infant regulatory function acts as a protective factor for later traits of autism spectrum disorder and attention deficit/hyperactivity disorder but not callous unemotional traits. J. Neurodev. Disord..

[105] Dackis M.N., Rogosch F.A., Cicchetti D. (2015). Child maltreatment, callous-unemotional traits, and defensive responding in high-risk children: An investigation of emotion-modulated startle response. Dev. Psychopathol..

[106] Elizur Y., Somech L.Y., Vinokur A.D. (2017). Effects of Parent Training on Callous-Unemotional Traits, Effortful Control, and Conduct Problems: Mediation by Parenting. J. Abnorm. Child Psychol..

[107] Short R.M.L., Adams W.J., Garner M., Sonuga-Barke E.J.S., Fairchild G. (2016). Attentional Biases to Emotional Faces in Adolescents with Conduct Disorder, Anxiety Disorders, and Comorbid Conduct and Anxiety Disorders. J. Exp. Psychopathol..

[108] McDonald S.E., Dmitrieva J., Shin S., Hitti S.A., Graham-Bermann S.A., Ascione F.R., Williams J.H. (2017). The role of callous/unemotional traits in mediating the association between animal abuse exposure and behavior problems among children exposed to intimate partner violence. Child Abus. Negl..

[109] Fanti K.A., Andershed H., Colins O.F., Sikki M. (2017). Stability and change in callous-unemotional traits: Longitudinal associations with potential individual and contextual risk and protective factors. Am. J. Orthopsychiatry.

[110] O’Kearney R., Salmon K., Liwag M., Fortune C.A., Dawel A. (2017). Emotional Abilities in Children with Oppositional Defiant Disorder (ODD): Impairments in Perspective-Taking and Understanding Mixed Emotions are Associated with High Callous–Unemotional Traits. Child Psychiatry Hum. Dev..

[111] Mozley M.M., Lin B., Kerig P.K. (2018). Posttraumatic Overmodulation, Callous–Unemotional Traits, and Offending Among Justice-Involved Youth. J. Aggress. Maltreat. Trauma.

[112] White S.F., Van Tieghem M., Brislin S.J., Sypher I., Sinclair S., Pine D.S., Hwang S., Blair R.J.R. (2016). Neural correlates of the propensity for retaliatory behavior in youths with disruptive behavior disorders. Am. J. Psychiatry.

[113] Waller R., Gardner F., Shaw D.S., Dishion T.J., Wilson M.N., Hyde L.W. (2015). Callous-Unemotional Behavior and Early-Childhood Onset of Behavior Problems: The Role of Parental Harshness and Warmth. J. Clin. Child Adolesc. Psychol..

[114] Cohen-Salmon C., Côté S., Fourneret P., Gasquet I., Guedeney A., Hamon M., Lamboy B., Le Heuzey M.-F., Michel G., Jean-Philippe R. (2017). Troubles des Conduites Chez L’enfant et L’adolescent Charles. Ph.D. Thesis.

[115] Kochanska G., Barry R.A., Stellern S.A., Bleness J.J.O. (2009). Early Attachment Organization Moderates the Parent—Child Mutually Coercive Pathway to Children ’ s Antisocial Conduct. Child Dev..

[116] Muratori P., Lochman J.E., Lai E., Milone A., Nocentini A., Pisano S., Righini E., Masi G. (2016). Which dimension of parenting predicts the change of callous unemotional traits in children with disruptive behavior disorder?. Compr. Psychiatry.

[117] Bedford R., Wagner N.J., Rehder P.D., Propper C., Willoughby M.T., Mills-Koonce R.W. (2017). The role of infants’ mother-directed gaze, maternal sensitivity, and emotion recognition in childhood callous unemotional behaviours. Eur. Child Adolesc. Psychiatry.

[118] Silva T.C., Graña J.L., González-Cieza L. (2014). Self-reported physical and emotional abuse among youth offenders and their association with internalizing and externalizing psychopathology: A preliminary study. Int. J. Offender Ther. Comp. Criminol..

[119] Horan J.M., Brown J.L., Jones S.M., Aber J.L. (2016). The Influence of Conduct Problems and Callous-Unemotional Traits on Academic Development Among Youth. J. Youth Adolesc..

[120] Kochanska G., Boldt L.J., Kim S., Yoon J.E., Philibert R.A. (2015). Developmental interplay between children’s biobehavioral risk and the parenting environment from toddler to early school age: Prediction of socialization outcomes in preadolescence. Dev. Psychopathol..

[121] Meffert H., Thornton L.C., Tyler P.M., Botkin M.L., Erway A.K., Kolli V., Pope K., White S.F., Blair R.J.R. (2018). Moderation of prior exposure to trauma on the inverse relationship between callous-unemotional traits and amygdala responses to fearful expressions: An exploratory study. Psychol. Med..

[122] Raschle N.M., Menks W.M., Fehlbaum L.V., Tshomba E., Stadler C. (2015). Structural and Functional Alterations in Right Dorsomedial Prefrontal and Left Insular Cortex Co-Localize in Adolescents with Aggressive Behaviour: An ALE Meta-Analysis. PLoS ONE.

[123] Roslyne Wilkinson H., Jones Bartoli A. (2021). Antisocial behaviour and teacher–student relationship quality: The role of emotion-related abilities and callous–unemotional traits. Br. J. Educ. Psychol..

[124] Ronald Bell G., Crothers L.M., Hughes T.L., Kanyongo G.Y., Kolbert J.B., Parys K. (2018). Callous-unemotional traits, relational and social aggression, and interpersonal maturity in a sample of behaviorally disordered adolescents. J. Appl. Sch. Psychol..

[125] White S.F., Cruise K.R., Frick P.J. (2009). Differential correlates to self-report and parent-report of callous-unemotional traits in a sample of juvenile sexual offenders. Behav. Sci. Law.

[126] Berg J.M., Lilienfeld S.O., Reddy S.D., Latzman R.D., Roose A., Craighead L.W., Pace T.W.W., Raison C.L. (2013). The Inventory of Callous and Unemotional Traits. Assessment.

[127] Forth A.E., Kosson D.S., Hare R.D. (2003). The Hare Psychopathy Checklist: Youth Version.

[128] Frick P.J. (2013). Clinical Assessment of Prosocial Emotions (Cape).

[129] Colins O.F., Andershed H., Frogner L., Lopez-Romero L., Veen V., Andershed A.-K. (2014). A New Measure to Assess Psychopathic Personality in Children: The Child Problematic Traits Inventory. J. Psychopathol. Behav. Assess..

[130] Garcia M., Rouchy E., Michel G. (2020). Les traits pré-psychopathiques chez l’enfant et l’adolescent: Aspects développementaux, structuraux et étiopathogéniques. Neuropsychiatr. Enfance. Adolesc..

[131] Andershed H., Colins O.F., Salekin R.T., Lordos A., Kyranides M.N., Fanti K.A. (2018). Callous-Unemotional Traits Only Versus the Multidimensional Psychopathy Construct as Predictors of Various Antisocial Outcomes During Early Adolescence. J. Psychopathol. Behav. Assess..

[132] Fanti K.A., Frick P.J., Georgiou S. (2009). Linking callous-unemotional traits to instrumental and non-instrumental forms of aggression. J. Psychopathol. Behav. Assess..

[133] Gao Y., Raine A., Venables P.H., Dawson M.E., Mednick S.A. (2010). Association of Poor Childhood Fear Conditioning and Adult Crime. Am. J. Psychiatry.

[134] Raine A. (2018). Antisocial Personality as a Neurodevelopmental Disorder. Annu. Rev. Clin. Psychol..

[135] Marsh A.A. (2013). What can we learn about emotion by studying psychopathy?. Front. Hum. Neurosci..

[136] Blair R.J.R. (2003). Facial expressions, their communicatory functions and neuro–cognitive substrates. Philos. Trans. R. Soc. Lond. Ser. B Biol. Sci..

[137] Puzzo I., Seunarine K., Sully K., Darekar A., Clark C., Sonuga-Barke E.J.S., Fairchild G. (2018). Altered White-Matter Microstructure in Conduct Disorder Is Specifically Associated with Elevated Callous-Unemotional Traits. J. Abnorm. Child Psychol..

[138] Armstrong K., Kimonis E.R. (2013). Parent-child interaction therapy for the treatment of Asperger’s disorder in early childhood: A case study. Clin. Case Stud..

[139] Foulkes L., Mccrory E.J., Neumann C.S., Viding E. (2014). Inverted Social Reward: Associations between Psychopathic Traits and Self-Report and Experimental Measures of Social Reward. PLoS ONE.

[140] Jones S., Cauffman E., Miller J.D., Mulvey E. (2006). Investigating Different Factor Structures of the Psychopathy Checklist: Youth Version: Confirmatory Factor Analytic Findings. Psychol. Assess..

[141] Longman T., Hawes D.J., Kohlhoff J. (2016). Callous–Unemotional Traits as Markers for Conduct Problem Severity in Early Childhood: A Meta-analysis. Child Psychiatry Hum. Dev..

[142] Raine A., Portnoy J., Liu J., Mahoomed T., Hibbeln J.R. (2015). Reduction in behavior problems with omega-3 supplementation in children aged 8-16 years: A randomized, double-blind, placebo-controlled, stratified, parallel-group trial. J. Child Psychol. Psychiatry Allied Discip..

[143] Waller R., Hyde L.W., Baskin-Sommers A.R., Olson S.L. (2017). Interactions between Callous Unemotional Behaviors and Executive Function in Early Childhood Predict later Aggression and Lower Peer-liking in Late-childhood. J. Abnorm. Child Psychol..

[144] García-Carmona M., Marín M.D., Aguayo R. (2019). Burnout syndrome in secondary school teachers: A systematic review and meta-analysis. Soc. Psychol. Educ..

[145] Lahey B.B. (2014). What we need to know about callous-unemotional traits: Comment on frick, ray, thornton, and kahn (2014). Psychol. Bull..

[146] Fragkaki I., Cima M., Granic I. (2018). The role of trauma in the hormonal interplay of cortisol, testosterone, and oxytocin in adolescent aggression. Psychoneuroendocrinology.

[147] Fairchild G., Hawes D.J., Frick P.J., Copeland W.E., Odgers C.L., Franke B., Freitag C.M., De Brito S.A. (2019). Conduct disorder. Nat. Rev. Dis. Prim..

[148] Allen J.L., Bird E., Chhoa C.Y. (2018). Bad Boys and Mean Girls: Callous-Unemotional Traits, Management of Disruptive Behavior in School, the Teacher-Student Relationship and Academic Motivation. Front. Educ..

[149] Fontaine N.M.G., Rijsdijk F.V., McCrory E.J.P., Viding E. (2010). Etiology of Different Developmental Trajectories of Callous-Unemotional Traits. J. Am. Acad. Child Adolesc. Psychiatry.

