# Supplementary material for: Role of Callous and Unemotional (CU) Traits on the Development of Youth with Behavioral Disorders: A Systematic Review
Source: Int J Environ Res Public Health. 2021 Apr 28;18(9):4712. doi: 10.3390/ijerph18094712 (PMC8125599; doi:10.3390/ijerph18094712)
Supplement: Supplementary file 1 [file ijerph-18-04712-s001.zip › ijerph-1168545-supplementary.pdf]

International Journal of
Environmental Research and Public Health

Table S1. Characteristics of the studies included in the review

First author, year	Country	N	% Boys	H[1]	Ctx	Adj	Int	Cg
Aghajani, 2017	NL	74	100	x			x	x
Bakker-Huvenaars, 2020	NL	114	100	x				x
Baroncelli, 2020	IT	301	49	x	x			
Bedford, 2019	UK	104	40	x				x
Bedford, 2017	USA	206		x	x			
Bird, 2019	UK	437	51	x		x		x
Breeden, 2015	USA	47	55	x				
Craig, 2019	EU/MC	245	70	x				
Crum, 2016	CA	1554	52	x	x	x		x
Dackis, 2015	USA	132	52	x	x	x	x	
Elizur, 2018	IL	209	78	x	x			
Elizur, 2017	IL	209	78	x	x			x
Erdogan, 2017	RO	51	45	x				x
Ezpeleta, 2017a	SP	622	79	x				x
Ezpeleta, 2017b	SP	320	51	x				
Fagan, 2017	USA	339	48	x			x	x
Fanti, 2017	CY	1200	47	x	x	x		x
Georgiou, 2019	CY	163	66	x				
Gonzalez-Madruga, 2020	EU/MC	200	49	x				
Graziano, 2019	USA	249	78	x				x
Grazioplene, 2020	USA	95	64	x				
Grotzinge, 2018	USA	891	49	x	x		x	
Hitti, 2019	USA	265	48	x			x	
Horan, 2016	USA	942	49	x	x			x
Kimonis, 2019	AU	23	87	x	x		x	
Kochanska, 2015	USA	82	55		x		x	
Levy, 2017	IL	67	100	x			x	
Masi, 2018	IT	144	89	x	x			
McDonald, 2017	USA	291	53	x	x			
Meffert, 2018	USA	72		x	x	x		
Mozley, 2018	USA	829	74	x		x	x	
Muratori, 2016	IT	126	90	x	x			
Naaijen, 2020	EU/MC	254	73	x				
O'Kearney, 2017	AU	124	64	x				
Raschle, 2018	EU/MC	189	43	x				
Ray, 2016	USA	1216	100	x				
Rizeq, 2020	CA	81	68	x				x
Rogers, 2019	EU/MC	298	46					x
Scavenius, 2019	DK	576	49		x			
Sethi, 2018	UK	95	100	x				x
Short, 2016a	UK	104	62	x				x
Short, 2016b	UK	99	61	x				
Sourander, 2016	FI	464	62		x			
Takahashi, 2020	UK	8958		x	x			
Thomson, 2019	UK	62	86	x		x		
Wall, 2016	CY	1366	47	x	x			x
Waller, 2015	USA	364	51	x	x			
Waller, 2018	USA	454	52	x	x		x	

Werhahn, 2020	CH/NL	207	73	x				
White, 2016	USA	56	57	x				
Wilkinson, 2020	UK	108	61	x	x			1
Winstanley, 2020	UK	145	77			x		1
K=52		25’823	65	47	22	8	10	19

[1] Health, Context, Adjustment, Interactions, Cognition

Table S2. Summary of study design, key methods and principal instruments

Ref. No	First author Year	Sample (Age mean)	Topic	Study type / design	Key methods / instruments
[69]	Aghajani 2017	N = 74 (16.96)	Connectivity of amygdala	Cross-sectional	Self-reports of CD, aggression, empathy, social responsiveness psychopathic traits and CU traits; fMRI. K-SADS-PL/ICU/BES/SRS/RPQ/YSR/YPI
[75]	Bakker-Huvenaars 2020	N = 114 (15.4)	Levels of oxytocin, cortisol, and testosterone	Cross-sectional	Caretaker and self-reports of diagnosis (TDI, ASD, ODD/CD) and CU traits; self-report of aggression; individually administered test of intelligence; salivary oxytocin, cortisol and testosterone. SCQ /DISC-IV/WISC-III/RPQ/ICU
[105]	Baroncelli 2020	N = 301 (12.96)	Student-teacher relationship	Longitudinal T1/T2 (6 months)	Self-report of CU traits and internalizing/externalizing problems; self-reports of student-teacher relationship quality and social preference. ICU/SDQ/STRQ
[104]	Bedford 2019	N = 79 (3,5)	Executive function as protective factors	Longitudinal T1/T5 (6 years)	Clinician rating of ASD ; parent reports of temperament (regulatory function, activity level), ASD, ADHD and CU traits. ADI-R/ADOS-2/SCQ/AOSI/IBQ-R/SRS/ICU/Conners 3
[106]	Bedford 2017	N = 206 (3,5)	Facial emotion recognition and maternal sensitivity	Longitudinal T1/T4 (6 years)	Measurement of multiple indices of mother-directed gaze, maternal sensitivity, children emotion recognition; parent report of CU traits. FFSFP/ACES/ICU/ASEBA
[7]	Bird 2019	N = 437 (12.50)	Performance in English, Science and Math grades	Cross-sectional	Teacher, parent and self-report of externalizing problems; self-report of CU traits; teacher assessment of academic performance. SPSRQ-C/ICU

[83]	Breeden 2015	N = 47 (14.37)	White matter integrity	Cross-sectional	Parent/guardian report of conduct problems; Parent/guardian and self-report of CU traits; individually administered test of intelligence ; fMRI face-emotion processing task; white matter microstructural integrity assessed using diffusion tensor imaging. K-BIT/ SDQ/CBCL/ICU
[99]	Craig 2019	N = 245 (13.2)	Structure and function fronto-amygdala	Cross-sectional	Clinician report of conduct problem and aggressive behavior; parent report of CU traits; individually administered test of intelligence; proton magnetic resonance spectroscopy. K-SADS/ICU/RPQ/WISC III/IV
[70]	Crum 2016	N = 1554 (8.13)	Compare children with ODD alone, CU alone, both and neither	Longitudinal T1/T2 (7 months)	Teacher reports of ADHD, ODD, conduct problems, CU traits, quality of student-teacher relationship (STR) and developmental impairment. ADS-IV/NSIC/STRS/IRS
[107]	Dackis 2015	N = 132 (10.36)	Effects of child maltreatment	Cross-sectional	Counselor report of CU traits and CD; self-report of antisocial behaviors and substance use; measures of physiological responses to visual stimuli (pleasant, neutral, and unpleasant images) using acoustic startle response (ASR). MCS/ICU/TRF/SRA
[108]	Elizur 2018	N = 209 (4.01)	Parent training's effects	Longitudinal T1/T2 (3 years)	Teacher report of CD ; parent reports of effortful control, CU traits, CD, parenting practices, parental distress, helplessness, parenting inefficacy; parental training. ECBI/CBQ/ICU/APQ/PSI-SF
[109]	Elizur 2017	N = 209 (4.01)	Parent training's effects	Longitudinal T1/T2 (3 years)	Parents reports of effortful control, CU traits, CD, parenting practices, parental distress, helplessness, parenting inefficacy; parental training. ECBI/CBQ/ICU/APQ/PSI-SF

[72]	Erdogan 2017	N = 51 (5.25)	Attentional orientation and emotions	Cross-sectional	Parent reports of child anxiety and temperament; teacher report of CU traits; parent and teacher report of emotional and behavioral problems (EBP); attentional orienting of emotional faces assessed by pictorial Dot-probe task. SCAS/ICU/CBCL/CBQ
[21]	Ezpeleta 2017a	N = 622 (3,77)	Co-occurrence with anxiety and ODD levels	Longitudinal T1/T6 (4 years)	Teacher reports of CU traits, ODD, temperament, executive function, aggressive behavior, social cognition deficit and irritability; parent reports of anxiety, EBP, emotion regulation, risk factors, parental practices, child sensitivity to reinforcement, child psychological disorder, adult psychopathology; assessment of attention function and response inhibition; clinician report of functional impairment. ICU/SDQ/CBCL
[71]	Ezpeleta 2017b	N = 320 (8,0)	Emotional recognizing	Cross-sectional	Teacher reports of ODD and CU traits; attention to emotion task; go/no-go task. SDQ/ICU
[84]	Fagan 2017	N = 339 (9,6)	Autonomic nervous system activity	Cross-sectional	Parent reports for aggressive behavior, rule breaking, narcissism, impulsivity, CU traits ; parent interview to assess ODD or CD symptoms; self-report of social adversity; measures of autonomic nervous system (ANS) activity, including heart rate variability (HRV), pre-ejection period (PEP), and heart rate using electrocardiogram. CBCL/APSD/ICU/DISC-IV
[110]	Fanti 2017	N = 1200 (9,38)	Developmental heterogeneity	Longitudinal T1/T3 (1 year)	Parent and self-reports of CU traits, CD, ADHD, narcissism, impulsivity; parent report of executive dysfunction; parent report of child self-regulation, empathy, and social competence; parent report of parental distress, parental involvement; self-report of perceived social support and school connectedness; teacher reports on academic performance. ICU/CSI-4/YI-4/APSD/ESQ/SEARS/PSI-SF/APQ/MSPSS

[101]	Georgiou 2019	N = 163 (4,95)	Affective and cognitive empathy (ASDs)	Cross-sectional	Parent report of CU traits, autistic traits, and empathy. SRS/GEM/ICU/ YPI
[86]	Gonzalez-Madruga 2020	N = 200 (15,44)	White matter microstructure of the extended limbic system	Cross-sectional	Parent and self-report of CU traits; structural connectivity using diffusion-weighted MRI. K-SADS/WASI/WISC/YPI/ICU
[111]	Graziano 2019	N = 249 (4,96)	Executive function and emotion regulation	Cross-sectional	Parent and teacher report of CU traits, executive function (EF), emotion regulation and conduct problems including ADHD, ODD and CD symptoms; self-report of self-regulation (EF); assessment of working memory skills; laboratory frustration tasks to elicit emotional distress and regulation. DBD/ICU/BRIEF-P/HTKS/ERC
[73]	Grazio-plene 2020	N = 95 (12,6)	White matter microstructure correlates	Cross-sectional	Parent report of aggressive behavior, CU traits, ADHD and anxiety; clinician interview for diagnosis; fiber density using fRMI. CBCL/ICU
[94]	Grotzinger 2018	N = 891 (15,91)	Links with testosterone and cortisol	Cross-sectional	Self-report of CU traits, rule breaking and aggression; self-report of pubertal development, peer deviance and prosociality, and parental monitoring; salivary testosterone, hair testosterone and cortisol. CBCL/ICU
[80]	Hitti 2019	N = 265 (12,27)	Effects of anger dysregulation	Longitudinal T1/T3 (6 months)	Self-report of beliefs supporting aggression, anger dysregulation, CU traits, externalizing behavior; teacher and self-report of physical aggression. BAFS/CAMS/ICU/SSIS-RS/PBFS-AR
[112]	Horan 2016	N = 942 (8,07)	Academic outcomes	Longitudinal T1/T2 (6 months)	Teacher report of STR quality, conduct problems, CU traits, social competence, aggression, responsibility and teaching stress; self-report of empathic attitudes; math and reading achievement obtained from the Department of Education. STRS/BASC-CP/BASC-A/ICU/CPS/CEAQ/SCS/RS/ITS

[87]	Kimonis 2019	N = 23 (4,5)	Parent-Child interaction therapy	Longitudinal T1-T2 (6 months)	Parent/caregiver report of CU traits, conduct problems, empathy and treatment acceptability, parental therapy ICU/ECBI/ASEBA/CBCL/GEM/TAI
[113]	Kochanska 2015	N = 82 (5,5)	Parenting environment	Longitudinal T1/T6 (8 years)	Positive, mutually responsive parenting (MRO) observed in naturalistic contexts; power-assertive discipline observed in laboratory room; children's anger proneness laboratory assessment; cheek swabs for genotyping; parent report of children's socialization outcomes and CU traits; self-report of attitude toward substance use and internalization of adult values. AVI/ICU
[79]	Levy 2017	N = 67 (16,2)	Oxytocin levels	Cross-sectional	Teacher and self-report of CU traits, social, emotional, and behavioral functioning ; past aggressive and delinquent behavior documented in personal files; salivary oxytocin. ICU/SDQ/BGLAS
[96]	Masi 2018	N = 144 (8,7)	Developmental trajectories	Longitudinal T1-T4 (6 years)	Clinical interview to assess psychiatric symptoms; self-report of CU traits and substance use; parent report of externalizing behavioral problems, parenting practices and use of mental health services; psycho-pharmacological intervention documented in clinical records. ICU/K-SADS-PL/WISC-III/APQ/CBCL/CSAP
[114]	McDonald 2017	N = 291 (9,07)	Exposure domestic violence and to animal maltreatment	Cross-sectional	Parent report of children's exposure to animal maltreatment; self-report of exposure to domestic violence; Maternal report of intimate partner violence (IPV); parent/caregiver report of CU traits and behavior problems. PTS/CEDV/CTS/ICU/CBCL
[115]	Meffert 2018	N = 72 (13,78)	Prior trauma exposure	Cross-sectional	Self-report of trauma; individually administered test of intelligence; parent report of internalizing and externalizing behavior, including levels of anxiety and post-traumatic stress disorder; parent and self-report of CU traits; morphed faces fMRI task; vignette procedure to assess social goals.

					CTQ/WASI-II/CBCL/ICU
[116]	Mozley 2018	N = 829 (16,07)	Trauma exposure and overmodulation	Cross-sectional	Self-report of trauma exposure, posttraumatic stress and CU traits; recidivism assessed by youth charges in juvenile justice records. PTSD-RI/ICU
[117]	Muratori 2016	N = 126 (10,0)	Positive and negative parenting practices	Longitudinal T1/T2 (2 years)	Self-report of CU traits; mother report of parenting practices and behavioral problems; clinician global functioning evaluation; clinician report of child global functioning improvement. K-SADS-PL/WISC-III/CBCL/ICU/APQ
[118]	Naaijen 2020	N = 254 (13,25)	Cortical and subcortical alterations	Cross-sectional	Structured diagnostic interviews to confirm clinical diagnoses of ODD, CD and possible comorbid ADHD; clinician report of aggressive/disruptive behavior; self-report of aggressive behavior; parent report of CU traits; measures of cortical volumes and cortical thickness (CT) and surface area using sMRI. K-SADS/CBCL/RPQ/ICU/WISC-III-IV
[119]	O'Kearney 2017	N = 124 (5,8)	Emotional process	Cross-sectional	Semi-structured diagnostic clinical interview, brief history-taking and developmental assessment with parent; child's emotional competencies, emotion perception, causal understanding of emotion assessment during interview; vignette procedure to assess emotion perspective-taking, ambivalent or mixed ambivalent emotional responses; clinical assessment of ODD; parent report of CU traits, empathy, emotional and behavioral problems; individually administered test of expressive vocabulary; parent report of emotional status (parental anxiety, depression and stress). ICU/DAKT/GEM/EVT0/DASS/SDQ
[74]	Raschle 2018	N = 189 (13,55)	Gray matter volume	Cross-sectional	Clinical diagnostic interview; clinician report of behavioral and emotional problems; individually administered test of intelligence; parent report of empathy; parent and self-report of CU traits; MPRAGE/FRMI /voxel-based morphometry. K-SADS-PL/CBCL/WASI/WISC-IV/ICU/YPI

[120]	Ray 2016	N = 1216 (15,29)	Role of neighborhood disorders	Cross-sectional	Self-report of delinquent behavior, abuse substance use, CU traits, impulse control, neighborhood disorder. WASI/SRO/ICU/WAI/SUAI/ND
[85]	Rizeq 2020	N = 81 (9,54)	Executive functions	Cross-sectional	Individually administered test of intelligence, cognitive flexibility, inhibitory control; structured interview to assess psychiatric disorders, symptoms, and level of impairment; parent reports of ADHD, ODD, CD, CU traits, executive functioning, and emotional and behavioral problems. K-BIT/ICU/CBCL/TMT
[82]	Rogers 2019	N = 298 (13,5)	White matter alterations	Cross-sectional	Clinical diagnostic interview to assess for CD and other common comorbid disorders; parent report of CU traits; individually administered test of intelligence; pubertal development assessment; emotional Go/No-Go task to assess for impulsivity; white matter alterations measurement using sMRI. K-SADS-PL/ICU/WASI
[121]	Scavenius 2019	N = 576 (14,5)	Functional Family Therapy	Cross-sectional	Parent and self-report emotional, and behavioral functioning, family functioning, youth school attendance, school performance, and drug and alcohol use; parent report of CU traits. SDQ/SCORE-15/ICU
[81]	Sethi 2018	N = 95 (14,4)	Empathic processing	Cross-sectional	Teacher and parent report of CD, CU traits; parent report of ADHD, anxiety, depression, and substance/alcohol abuse symptom; individually administered test of intelligence; self-report of alcohol and drug use disorder; parent report of youth mood disorders; vignette procedure to assess affective introspection; fMRI SDQ/CASI-CD/ICU/WASI
[122]	Short 2016a	N = 104 (16,6)	Attentional biases to emotional faces	Cross-sectional	Clinical interview to assess for CD and ODD; self-report of CU traits, anxiety and depressive symptoms; visual-probe task to assess attention to emotion. K-SADS-PL/ICU/STAI/HADS

[123]	Short 2016b	N = 99 (16,6)	Comorbid anxiety and emotion recognition	Cross-sectional	Clinical interview to assess for CD and ODD; self-report of CU traits, anxiety and depressive symptoms; assessment of social disadvantage; facial identity recognition and facial emotion recognition tasks. K-SADS-PL/ICU/STAI/HADS/NES/BFRT
[124]	Sourander 2016	N = 464 (4,5)	Internet-assisted parent training intervention	Longitudinal T0/T2 (1 year)	Parent report of conduct problems, externalizing behavior, CU traits ; depression, anxiety, stress and satisfaction; parent training. CBCL/ICU
[88]	Takahashi 2020	N = 8958 (11,08)	Genetic and environmental influences	Longitudinal T1/T4 (9 years)	Parent report of CU traits; phenotype tests (twin pairs DZ-MZ). ICU/SDQ/APSD
[77]	Thomson 2020	N = 62 (12,54)	Emotions and (para) sympathetic reactivity	Cross-sectional	Self-report of CU traits, arousal and emotional reactivity; parent report of conduct problems and prosocial behavior; emotional and physiological reactivity to fear through 3D roller coaster simulation video; fear reactivity and physiological data assess by electrocardiogram. ICU/SDQ
[76]	Wall 2016	N = 1366 (9,38)	Characteristics of children with ou without CU	Longitudinal T1/T3 (1 year)	Parent report of CU traits, conduct problems, impulsivity, executive control; parent and self-report of parental involvement and positive parenting; self-report of school connectedness. ICU/CSI-4/APSD/SEARS/ESQ/APQ/MSPSS
[125]	Waller 2015	N = 364 (3,0)	Parental harshness	Longitudinal T1/T3 (2 years)	Parent report of CU traits, behavior problems, parenting practices, warmth in the parent–child relationship; observed harsh parenting assessed by coded videotaped family tasks; parental warmth coded from 5-min parental speech samples. ECBI/ACRS/ICU/FAARS
[36]	Waller 2018	N = 454 (7,8)	Parenting and behavior problems	Cross-sectional	Parent report of parental harshness, parental warmth, aggression, CU traits, interviews. PEQ/ICU/STAB
[126]	Werhahn 2020	N = 207 (13,3)	Resting state functional connectivity (brain)	Cross-sectional	Semi-structured diagnostic clinical interview, including ADHD; self-report of aggression; parent report of CU traits; measure of functional

					connectivity using a seed-to- voxel approach (fMRI). ICU/K-SADS-PL/CBCL/WASI/YSR/TRF
[127]	White 2016	N = 56 (14,74)	Neural correlates	Cross-sectional	Parent report of CU traits, aggression ; ultimatum game (social fairness game) task during fMRI ; fMRI. ICU/RPARS
[128]	Wilkinson 2020	N = 108 (8,88)	Teacher–Student Relationship	Cross-sectional	Teacher report of antisocial behavior, CU traits, emotion-related behavior and STR quality. BASC/ICU/ERC/STRS
[78]	Winstanley 2020	N = 145 (14,6)	Developmental language disorders	Cross-sectional	Individually administered test of intelligence, language ability; self-report of CU traits; measure of adversity constructed from the data extracted from the youth justice service files; prior offending and age at first offence obtained via detailed scrutiny of departmental files in each youth justice setting; assessment of area-level deprivation. WASI/ICU/CELF-4/IMD

Note: **ACES**: Assessment of Children's Emotional Skills; **ACRS**: Adult Child Relationship Scale; **ADI-R**: Autism Diagnostic Interview—Revised; **ADOS-2** : Autism Diagnostic Observation Schedule—Second Edition**;** Ag-AgCI **ADS-IV**:Assessment of Disruptive Symptoms–DSM-IV Version; **AOSI**: Autism Observation Scale for Infants; **APQ**:Alabama Parenting Questionnaire; **APSD**: Antisocial Process Screening Device; **ASEBA**: Achenbach System of Empirically Based Assessment; **AVI**: Adolescent Values Inventory; **BAFS**: Beliefs About Fighting Scale; **BASC**: Behavioral Assessment System for Children **BASC-A**: Behavioral Assessment System for Children—Aggression; **BASC-CP**: Behavioral Assessment System for Children—Conduct Problems; **BES:** Basic Empathy Scale; **BFRT**: Benton Facial Recognition Test; **BGLAS:** Brown-Goodwin Lifetime Aggression Scale**; BRIEF-P**: Behavior Rating Inventory of Executive Function–Preschool Version; **CAMS**: Children's Anger Management Subscale; **CASI-CD**: Child and Adolescent Symptom Inventory–Conduct Disorder; **CBCL**: Child Behavior Checklist; **CBQ**: Child Behavioral Questionnaire; **CEAQ**: Children's Empathic Attitudes Questionnaire; **CEDV**: Children's Exposure to Domestic Violence; **CELF-4**: Clinical Evaluation of Language Fundamentals; **Conners 3** : ADHD scale; **CSI-4**: Checkmate plus Child Symptom Inventory for Parents-4; **CPS**: Childhood Psychopathy Scale; **CSAP**: Substance Abuse Prevention Student Survey; **CTQ**: Childhood Trauma Questionnaire; **CTS**: Conflict Tactic Scale; **DAKT**: Denham Affective Knowledge Tests; **DASS**: Depression, Anxiety and Stress Scale; **DBD**: Disruptive Behavior Disorders Rating Scale; **DICS-IV:** Diagnostic Interview Schedule for Children; **ECBI:** Eyberg child behavior inventory; **ERC**: Emotion Regulation Checklist; **ESQ**: Executive Skills Questionnaire; **EVT0**: Expressive Vocabulary Test; **FAARS**: Positive subscale of the Family Affective Attitudes Rating Scale; **FFSFP**: Face-to-Face Still-Face Paradigm; **fMRI**: functional Magnetic Resonance Imaging; **GEM**: Griffith Empathy Measure; **K-BIT**: Kaufman Brief Intelligence Test; **K-SADS-PL**: Kiddie-Schedule; **IBQ-R**: Infant Behavior Questionnaire—Revised; **HADS**: Hospital Anxiety and Depression Scales**; HTKS**: Head-Toes-Knees-Shoulders Task; **ICU**: Inventory of Callous and Unemotianal traits; **IMD**: Index of Multiple

Deprivation; **IRS**: Impairment Rating Scale; **ITS**: Index of Teaching Stress; **MCS**: Maltreatment Classification System; **MSPSS**: Multidimensional Scale of Perceived Social Support; **ND**: Neighborhood disorder; **NES**: Neighbourhood Envi-ronment Scale; **NSIC**: Nova Scotia Modified IOWA Conners; **PBFS-AR**: Problem Behavior Frequency Scale Adolescent Report; PEQ: Parental Environment Questionnaire; **PSI-SF**: Parenting Stress Index-Short Form; **PTS**: Pet Treatment Survey; **PTSD-RI**: Posttraumatic Stress Disorder Reaction Index; **RPARS**: Reactive/Proactive Aggression Rating Scale**; RPQ**: Reactive-Proactive Aggression Questionnaire; **RS**: Responsibility Scale; **SCAS**: Spence Preschool Anxiety Scale; **SEARS**: Social-Emotional Assets and Resilience scale; **SCORE-15**: Systemic Clinical Outcome and Routine Evaluation; **SCS**: Social Competence Scale; **SCQ**: Social Communication Questionnaire; **SDQ**: Strength and Difficulties Questionnaire; **SPSRQ-C**: Sensitivity to Punishment and Sensitivity to Reward Questionnaire; **SRA**: Self-Reported Antisocial Behavior Scale; **SRO**: Self-report of Offending Scale; **SRS**: Social Responsiveness Scale; **SSIS-RS**: Social Skills Improvement System; **STAB**: Subtypes of Antisocial Behavior Questionnaire; **STAI**: State-Trait Anxiety Inventory; **STRS**: Student-Teacher Relationship Scale; **STRQ**: Student-Teacher Relationship Questionnaire; **SUAI**: Substance Use/Abuse Inventory; **TAI**: Therapy Attitude Inventroy; **TMT**: Trail making Test; **TRF**: Teacher Report Form; **WAI**: Weinberger Adjustment Inventory; **WASI**: Wechsler Abbreviated Scale of Intelligence **WISC:** Wechsler Intelligence Scale for Children; **YI-4**: Checkmate plus Youth's Inventory-4; **YPI**: Youth Psychopathic Traits Inventory; **YSR**: Youth Self-Report.

Table S3. Summary of results – Mental Health

Ref. No	First author Year	Sample (Age mean)	Topic	Summary of results
[69]	Aghajani 2017	N = 74 (16.96)	Connectivity of amygdala	Significant differences on ODD/CD found between groups with or without CU traits (more emotional dysregulation). No links with internalized disorders
[75]	Bakker-Huvenaars 2020	N = 114 (15.4)	Levels of oxytocin, cortisol, and tes-tosterone	ODD linked with high score CU traits
[105]	Baroncelli 2020	N = 301 (12.96)	Student-teacher relationship	Significant links with externalized behavioral disorders were found at T2. No links with internalized disorders
[104]	Bedford 2019	N = 79 (3,5)	Executive function as protective fac-tors	Correlations found between children at high risk of ASD or ADHD and CU traits
[106]	Bedford 2017	N = 206 (3,5)	Facial emotion recognition and maternal sensitiv-ity	Significant effect of emotion recognition in predicting CU traits
[7]	Bird 2019	N = 437 (12.50)	Performance in English, Science and Math grades	CU traits related to externalizing disorders
[83]	Breeden 2015	N = 47 (14.37)	White matter in-tegrity	The severity of externalizing behavior is not related to white-matter integrity after controlling for CU traits

[99]	Craig 2019	N = 245 (13.2)	Structure and function fronto-amygdala	Significant links with externalized behavioral disorders and CU traits
[70]	Crum 2016	N = 1554 (8.13)	Compare children with ODD alone, CU alone, both and neither	Children with CU Significant links with externalized behavioral disorders (ODD/CD) and CU traits
[107]	Dackis 2015	N = 132 (10.36)	Effects of child maltreatment	Significant links with externalized behavioral disorders and CU traits
[108]	Elizur 2018	N = 209 (4.01)	Parent training's effects	Significant links with externalized behavioral disorders and CU traits
[109]	Elizur 2017	N = 209 (4.01)	Parent training's effects	Significant links between externalized behavioral disorders and CU traits
[72]	Erdogan 2017	N = 51 (5.25)	Attentional orientation and emotions	Significant links with externalized behavioral disorders (ODD) and CU traits<br>Results indicated significant interaction effects between ODD-related problems and CU, as well as between CU and anxiety
[21]	Ezpeleta 2017a	N = 622 (3,77)	Co-occurrence with anxiety and ODD levels	Significant links between CU traits and externalized behavioral disorders (ODD) and anxiety and/or emotional disorders
[71]	Ezpeleta 2017b	N = 320 (8,0)	Emotional recognizing	Children with ODD and CU are less accurate in recognizing emotion and deficits in emotion processing
[84]	Fagan 2017	N = 339 (9,6)	Autonomic nervous system activity	Significant links between externalized behavioural disorders and CU traits
[110]	Fanti 2017	N = 1200 (9,38)	Developmental heterogeneity	Exhibit high and stable levels of conduct problems, attention-deficit/hyperactivity disorder symptoms, impulsivity
[101]	Georgiou 2019	N = 163 (4,95)	Affective and cognitive empathy (ASDs)	CU traits were negatively related with both cognitive and affective empathy in ASD children<br>CU traits negatively predicted affective empathy only among girls high on autistic traits.
[111]	Graziano 2019	N = 249 (4,96)	Executive function and emotion regulation	Poorer emotional regulation was associated with greater levels of CU behaviors.
[80]	Hitti 2019	N = 265 (12,27)	Effects of anger dysregulation	Significant links between externalized behavioral disorders and CU traits<br>Significant links between anger dysregulation and CU traits
[112]	Horan 2016	N = 942 (8,07)	Academic outcomes	CU traits are associated with ODD and lack of empathy

[87]	Kimonis 2019	N = 23 (4,5)	Parent-Child interaction therapy	Intervention produced increases in empathy in child with CU traits
[79]	Levy 2017	N = 67 (16,2)	Oxytocin levels	CU traits are associated with ODD/CD
[96]	Masi 2018	N = 144 (8,7)	Developmental trajectories	CU traits are associated with externalizing and internalizing disorders
[114]	McDonald 2017	N = 291 (9,07)	Exposure to domestic and animal violence	CU traits are associated with externalizing and internalizing disorders
[116]	Mozley 2018	N = 829 (16,07)	Trauma exposure and overmodulation	An association between interpersonal trauma exposure and persistent offending exhibited an indirect effect through overmodulation and CU traits
[117]	Muratori 2016	N = 126 (10,0)	Positive and negative parenting practices	CU traits are associated with externalizing disorders
[119]	O'Kearney 2017	N = 124 (5,8)	Emotional process	ODD boys with high levels of CU traits showed deficits in emotion perspective-taking and in understanding ambivalent emotions.
[85]	Rizeq 2020	N = 81 (9,54)	Executive functions	Correlations between CU traits and ODD/CD
[81]	Sethi 2018	N = 95 (14,4)	Empathic processing and cortex	Correlations between CU traits and ODD/CD/internalizing disorders<br>Findings provide limited and ultimately equivocal evidence for altered affective introspection regarding others in CP/HCU, and altered affective introspection for own emotions in CP/LCU
[122]	Short 2016a	N = 104 (16,6)	Attentional biases to emotional faces	Within the CD-only and comorbid CD+anxiety disorder groups, there were no significant effects or interactions involving CU traits.
[123]	Short 2016b	N = 99 (16,6)	Comorbid anxiety and emotion recognition	CD and CD/anxiety have higher scores of CU traits<br>CU+ differ in the recognition of fear, joy and disgust

[77]	Thomson 2020	N = 62 (12,54)	Emotions and (para) sympathetic reactivity	Higher CD in group with CU<br>Youths with CU traits self-reported that they felt more in control after the fear induction. They displayed a physiological and emotional profile suggesting they maintained control during fear induction
[76]	Wall 2016	N = 1366 (9,38)	Characteristics of children with ou without CU	The high-CU group had significantly lower hyperactivity-impulsivity
[125]	Waller 2015	N = 364 (3,0)	Parental harshness	Correlation between CU traits and ODD/CD
[128]	Wilkinson 2020	N = 108 (8,88)	Teacher–Student Relationship	CU traits are linked to lower emotion understanding/empathy. higher emotion lability

3.4. Neurobiological markers and CU traits (physical Health)

Table S4. Summary of results – Neurobiological Markers

Ref. No	First author Year	Sample (Age mean)	Topic	Summary of results
[69]	Aghajani 2017	N = 74 (16.96)	Connectivity of amygdala	Relative to CD/CU showed abnormally increased BLA connectivity with a cluster that included both dorsal and ventral portions of the anterior cingulate and medial prefrontal cortices, along with posterior cingulate, sensory associative, and striatal regions.<br>Connectivity changes coincide with local hypotrophy of BLA and CMA subregions (without being statistically correlated) and were associated to more severe CU symptoms.
[75]	Bakker-Huvenaars 2020	N = 114 (15.4)	Levels of oxytocin, cortisol, and testosterone	Higher levels of CU traits were related to higher levels of cortisol and testosterone
[83]	Breeden 2015	N = 47 (14.37)	White matter integrity	Both CU traits and externalizing behaviors are negatively correlated with white-matter integrity in bilateral uncinate fasciculus and stria terminalis/fornix, simultaneously modeling both variables revealed that these effects are driven by CU traits.
[99]	Craig 2019	N = 245 (13.2)	Structure and function fronto-amygdala	CU traits were associated with increased anterior cingulate cortex (ACC) glutamate

[107]	Dackis 2015	N = 132 (10.36)	Effects of child maltreatment	Results indicated differential physiological responses for maltreated and non-maltreated children based on CU traits including a pathway of hypo arousal for non-maltreated /high CU children that differed markedly from a more normative physiological trajectory for maltreated/high CU children
[84]	Fagan 2017	N = 339 (9,6)	Autonomic nervous system activity	low resting heart rate but not pre-ejection period or heart rate variability, mediated the relationship between social adversity and antisocial behavior in males only.
[86]	Gonzalez-Madruga 2020	N = 200 (15,44)	White matter microstructure of the extended limbic system	Positive correlation between CD and right retrosplenial cingulum hindrance-modulated orientational anisotropy. There were no other significant correlations between CD, ADHD symptoms, overall psychopathic traits, the subdimensions of psychopathy, CU traits
[73]	Grazioplene 2020	N = 95 (12,6)	White matter microstructure correlates	Co-occurring CU traits and anxiety did not moderate the association between aggression and white matter density; Results reveal lower fiber density in a cluster of limbic and cortical pathways, including the inferior fronto-occipital fasciculus, fornix, middle cerebellar peduncle, and superior thalamic radiations, and had higher fiber density in the corpus callosum.
[94]	Grotzinger 2018	N = 891 (15,91)	Links with testosterone and cortisol	Salivary testosterone was not associated with aggression. Hair testosterone significantly predicted increased aggression, particularly at low levels of hair cortisol in children with CU
[79]	Levy 2017	N = 67 (16,2)	Oxytocin levels	Significant effect of conduct disorder and oxytocin on ICU-TR but not ICU-SR
[115]	Meffert 2018	N = 72 (13,78)	Prior trauma exposure	Significant CU traits-by-trauma exposure interaction was observed within right amygdala. Pathophysiology associated with CU traits differs in youth as a function of prior trauma exposure.
[118]	Naaijen 2020	N = 254 (13,25)	Cortical and subcortical alterations	Proactive aggression was negatively associated with amygdala volume, while reactive aggression was negatively associated with insula volume. No associations were found with CU traits or ADHD symptomatology. Study showed negative associations between reactive aggression and volumes in a region involved in threat responsivity and between proactive aggression and a region linked to empathy.

[74]	Raschle 2018	N = 189 (13,55)	Gray matter volume	CU traits have a neuroanatomical correlate within typically developing boys, but not girls : significant positive correlation between CU traits and bilateral anterior insula volume in boys
[82]	Rogers 2019	N = 298 (13,5)	White matter alterations	Youths with CD exhibited higher axial diffusivity in the corpus callosum and lower radial diffusivity and mean diffusivity in the anterior thalamic radiation relative to TD youths
[81]	Sethi 2018	N = 95 (14,4)	Empathic processing and cortex	Analyses revealed no significant differences of activation in the anterior insula, anterior cingulate cortex / medial prefrontal cortex, and occipital cortex as significantly activated
[88]	Takahashi 2020	N = 8958 (11,08)	Genetic and environmental influences	Individual differences in the baseline level of CU traits were highly heritable (76.5%), while the heritability of the developmental course of CU traits was moderate (43.6%).
[77]	Thomson 2020	N = 62 (12,54)	Emotions and (para) sympathetic reactivity	Adolescents with high callous-unemotional traits showed coactivation of the sympathetic and parasympathetic nervous system.
[126]	Werhahn 2020	N = 207 (13,3)	Resting state functional connectivity (brain)	Callous-unemotional traits were linked to distinct hyper-connectivities to frontal, parietal, and cingulate areas.
[127]	White 2016	N = 56 (14,74)	Neural correlates	Relative to the other groups, youths with DBD and low levels of callous-unemotional traits showed greater increases in activation of basic threat circuitry when punishing others and dysfunctional down-regulation of the ventromedial prefrontal cortex during retaliation. Relative to healthy youths, all youths with DBD showed reduced amygdala-ventromedial prefrontal cortex connectivity during high provocation

Table S5. Summary of results – Family and School Context

Ref. No	First author Year	Sample (Age mean)	Topic	Summary of results
[105]	Baroncelli 2020	N = 301 (12.96)	Student-teacher relationship	Levels of affiliation with teachers are negatively related to levels of CU traits in students with low levels of social preference among peers

[106]	Bedford 2017	N = 206 (3,5)	Facial emotion recognition and maternal sensitivity	infants' mother directed gaze and maternal sensitivity are not significant, but their interaction significantly predicted CU traits.
[70]	Crum 2016	N = 1554 (8.13)	Compare children with ODD alone, CU alone, both and neither	Children with CD/CU had the highest conflict and lowest closeness with teachers at the start of the school year and the greatest end-of- year impairment, higher conflict and lower closeness with teachers at the school year's start were associated with greater end-of-the-year impairment
[107]	Dackis 2015	N = 132 (10.36)	Effects of child maltreatment	Experience and biology may influence the development of antisocial behavior in youth and highlight potential avenues for intervention
[108]	Elizur 2018	N = 209 (4.01)	Parent training's effects	Significant effects of parenting mediated treatment on both EC and CU traits
[109]	Elizur 2017	N = 209 (4.01)	Parent training's effects	Significant and positive effects of the intervention on the family context and the parent self-efficacy
[110]	Fanti 2017	N = 1200 (9,38)	Developmental heterogeneity	Experience low parental involvement and high parental distress report low peer support and school connectedness
[94]	Grotzinger 2018	N = 891 (15,91)	Links with testosterone and cortisol	High levels of CU traits linked to lack of parental monitoring
[112]	Horan 2016	N = 942 (8,07)	Academic outcomes	CU traits are associated with lower quality student–teacher relationships
[87]	Kimonis 2019	N = 23 (4,5)	Parent-Child interaction therapy	Family intervention produced decreases in child conduct problems and CU traits
[113]	Kochanska 2015	N = 82 (5,5)	Parenting environment	Links between CU traits Variations in positive parenting predicted cooperation with monitoring and negative attitude toward substance use; variations in power-assertive parenting predicted internalization of adult values and callous–unemotional traits.
[96]	Masi 2018	N = 144 (8,7)	Developmental trajectories	Positive parenting does not predict CU according to the model
[114]	McDonald 2017	N = 291 (9,07)	Exposure to domestic and animal violence	Effects of animal maltreatment exposure on externalizing problems are mediated through callousness

[115]	Meffert 2018	N = 72 (13,78)	Prior trauma exposure	Significant link between prior trauma exposure and CU traits
[117]	Muratori 2016	N = 126 (10,0)	Positive and negative parenting practices	No significant cross-lagged path was found between negative parenting and CU traits
[121]	Scavenius 2019	N = 576 (14,5)	Functional Family Therapy	Significant effect of FFT : Girls had significantly greater improvements in peer problems and family functioning, and boys benefited more in increased liking of school
[124]	Sourander 2016	N = 464 (4,5)	Internet-assisted parent training intervention	Results reveals the effectiveness and feasibility of an Internet-assisted parent training intervention offered for parents of preschool children with disruptive behavioral
[88]	Takahashi 2020	N = 8958 (11,08)	Genetic and environmental influences	Nonshared environment made a modest contribution to the baseline level of CU traits (21.7%)
[76]	Wall 2016	N = 1366 (9,38)	Characteristics of children with ou without CU	Parenting less maternal involvement and less connectedness to school
[125]	Waller 2015	N = 364 (3,0)	Parental harshness	Correlation between CU traits and ODD/CD<br>Twin differences in parental harshness were related to differences in both aggression and CU traits, such that the twin who received harsher parenting had higher aggression and more CU traits.<br>Differences in parental warmth were related to differences in CU traits, such that the twin receiving warmer parenting presented lower CU traits
[36]	Waller 2018	N = 454 (7,8)	Parenting and behavior problems	CU behavior moderated crosssectional correlations between parent-reported and observed warmth and child behavior problems.
[128]	Wilkinson 2020	N = 108 (8,88)	Teacher–Student Relationship	CU traits are associated with higher teacher–student closeness

Table S6. Summary of results – Social Adjustment

Ref. No	First author Year	Sample (Age mean)	Topic	Summary of results

[7]	Bird 2019	N = 437 (12.50)	Performance in English, Science and Math grades	Children with CU traits have less concern for consequences of aggression including anticipated feelings of remorse, disciplinary action, or victim distress
[110]	Fanti 2017	N = 1200 (9,38)	Developmental heterogeneity	Score lower social competence and self-regulation
[94]	Grotzinger 2018	N = 891 (15,91)	Links with testos-terone and cortisol	High levels of CU traits linked increased aggression
[80]	Hitti 2019	N = 265 (12,27)	Effects of anger dysregulation	Significant links with increased aggression
[113]	Kochanska 2015	N = 82 (5,5)	Parenting environment	Children with CD/CU who received optimal parenting had a more adaptive outcome than their low-risk peers (attitude toward substance)
[115]	Meffert 2018	N = 72 (13,78)	Prior trauma exposure	Significant link between CU traits and social adjustment (delinquency) by trauma exposure influence
[116]	Mozley 2018	N = 829 (16,07)	Trauma exposure and overmodulation	Overmodulation is associated with CU traits which are in turn associated with recidivism
[77]	Thomson 2020	N = 62 (12,54)	Emotional reactivity and (para) sympathetic reactivity	Youths with CU traits self-reported more antisocial behaviours

Table S7. Summary of results – Social interactions and social roles dimension

Ref. No	First author Year	Sample (Age mean)	Topic	Summary of results
[69]	Aghajani 2017	N = 74 (16.96)	Connectivity of amygdala	Significant difference in substance abuse
[107]	Dackis 2015	N = 132 (10.36)	Effects of child maltreatment	Children with high CU linked with elevated antisocial behavior increased aggressive behavior and delinquent acts (environmental influence)
[84]	Fagan 2017	N = 339 (9,6)	Autonomic nerv-ous system activ-ity	Significant links with antisocial behavior (females)
[94]	Grotzinger 2018	N = 891 (15,91)	Links with testos-terone and cortisol	High levels of CU traits linked to high levels of rule breaking peer deviance

[80]	Hitti 2019	N = 265 (12,27)	Effects of anger dysregulation	Significant correlations examining the relations between more global beliefs supporting aggression and aggression in peer and dating contexts
[87]	Kimonis 2019	N = 23 (4,5)	Parent-Child interaction therapy	By 3 months posttreatment, 75% of treatment completers no longer showed clinically significant conduct problems relative to 25% of dropouts
[113]	Kochanska 2015	N = 82 (5,5)	Parenting environment	Parent report of children's socialization outcomes and CU traits; internalization of parent' values have significant effects toward substance use
[79]	Levy 2017	N = 67 (16,2)	Oxytocin levels	Significant effect CU traits on misadaptation (aggression)
[116]	Mozley 2018	N = 829 (16,07)	Trauma exposure and overmodulation	Significant correlation between CU traits and aggression
[125]	Waller 2015	N = 364 (3,0)	Parental harshness	Correlation between CU aggressivity

Table S8. Summary of results – Cognitive dimension

Ref. No	First author Year	Sample (Age mean)	Topic	Summary of results
[69]	Aghajani 2017	N = 74 (16.96)	Connectivity of amygdala	Attention-emotion-interactions are perturbed, which could bias salience processing and associative learning in youth with CD/CU
[104]	Bedford 2019	N = 79 (3,5)	Executive function as protective factors	Infant with irregular executive functions (EF) at T1 are associated with CU traits at T2
[7]	Bird 2019	N = 437 (12.50)	Performance in English, Science and Math grades	CU traits are significantly related to lower English, Math and Science grades when controlling for age, gender, sociodemographic disadvantage (lower Science grades for boys only not for girls)
[70]	Crum 2016	N = 1554 (8.13)	Compare children with ODD alone, CU alone, both and neither	ADHD with CU traits are robustly associated with end-of-the-year impairment (EF)
[109]	Elizur 2017	N = 209 (4.01)	Parent training's effects	Significant links between EF and traits CU

[72]	Erdogan 2017	N = 51 (5.25)	Attentional orientation and emotions	Temperamental self-regulation is negatively related to CU traits and ODD-related problems
[21]	Ezpeleta 2017a	N = 622 (3,77)	Co-occurrence with anxiety and ODD levels	Results show deficits in executive functioning, attentional process.
[84]	Fagan 2017	N = 339 (9,6)	Autonomic nervous system activity	Significant links between CU traits and impulsivity (EF)
[110]	Fanti 2017	N = 1200 (9,38)	Developmental heterogeneity	Lower scores on academic performance, EF
[111]	Graziano 2019	N = 249 (4,96)	Executive function and emotion regulation	Children with CU/high- were rated as having significantly poorer ER and EF compared to other groups
[112]	Horan 2016	N = 942 (8,07)	Academic outcomes	CU traits are associated with worse performance on standardized math and reading exams over and above the effects of conduct problems.
[119]	O'Kearney 2017	N = 124 (5,8)	Empathic processing and cortex	Impairments in emotion perceptive-taking and understanding mixed emotions in ODD boys are associated with the presence of a high level of CU
[85]	Rizeq 2020	N = 81 (9,54)	Executive functions	CU traits and parent ratings of executive dysfunction were uniquely correlated with elevated conduct problems and oppositional and defiant behavior
[81]	Sethi 2018	N = 95 (14,4)	Empathic processing	No difference between groups regarding IQ
[123]	Short 2016b	N = 99 (16,6)	Comorbid anxiety and emotion recognition	CD was associated with a generalised impairment in emotion recognition (EF)
[76]	Wall 2016	N = 1366 (9,38)	Characteristics of children with ou without CU	The high-CU group had significantly executive functioning deficits
[127]	White 2016	N = 56 (14,74)	Neural correlates	Ventromedial prefrontal cortex responsiveness and ventromedial prefrontal cortex-amygdala connectivity were related to patients' retaliatory propensity (behavioral responses during the task) and parent-reported reactive aggression.

[128]	Wilkinson 2020	N = 108 (8,88)	Teacher–Student Relationship	CU traits are associated with bad emotional regulation (FE)
[78]	Winstanley 2020	N = 145 (14,6)	Developmental language disorders	Young with developmental language disorder are more than twice as likely to reoffend than their unaffected offending peers. The elevated risk of reoffending was not explained by nonverbal IQ, deprivation score, the presence of a neurodevelopmental disorder or callous–unemotional traits.
